# The Transition from Cancer “omics” to “epi-omics” through Next- and Third-Generation Sequencing

**DOI:** 10.3390/life12122010

**Published:** 2022-12-02

**Authors:** Konstantina Athanasopoulou, Glykeria N. Daneva, Michaela A. Boti, Georgios Dimitroulis, Panagiotis G. Adamopoulos, Andreas Scorilas

**Affiliations:** Department of Biochemistry and Molecular Biology, Faculty of Biology, National and Kapodistrian University of Athens, 15701 Athens, Greece

**Keywords:** massive parallel sequencing, epigenomics, epitranscriptomics, epiproteomics, cancer genomics, DNA methylation, RNA sequencing, pharmacogenomics

## Abstract

Deciphering cancer etiopathogenesis has proven to be an especially challenging task since the mechanisms that drive tumor development and progression are far from simple. An astonishing amount of research has revealed a wide spectrum of defects, including genomic abnormalities, epigenomic alterations, disturbance of gene transcription, as well as post-translational protein modifications, which cooperatively promote carcinogenesis. These findings suggest that the adoption of a multidimensional approach can provide a much more precise and comprehensive picture of the tumor landscape, hence serving as a powerful tool in cancer research and precision oncology. The introduction of next- and third-generation sequencing technologies paved the way for the decoding of genetic information and the elucidation of cancer-related cellular compounds and mechanisms. In the present review, we discuss the current and emerging applications of both generations of sequencing technologies, also referred to as massive parallel sequencing (MPS), in the fields of cancer genomics, transcriptomics and proteomics, as well as in the progressing realms of epi-omics. Finally, we provide a brief insight into the expanding scope of sequencing applications in personalized cancer medicine and pharmacogenomics.

## 1. Introduction

Almost 40 years have passed since the term genomics unexpectedly came into existence, paving the way for the emergence of omics and thus the beginning of a new chapter in biological sciences [1]. Dedicated to the in-depth analysis of a broad range of biomolecules (e.g., DNA, RNA, proteins and metabolites), the term “omics” represents fields of study that enable the exploration of biological systems at different layers, ranging from genome to proteome and even metabolome, hence providing a better understanding of the unparalleled complexity that surrounds the structure and molecular mechanisms of cells. For instance, genomics is defined by the study of the complete set of DNA of living organisms and the detection of its variations, while transcriptomics focuses on the comprehensive profiling and quantification of gene expression in various developmental stages and physiological conditions [2]. On the other hand, proteomics aims at the characterization of the cellular protein expression profile and abundance at a specific sample, metabolomics studies small molecules arising from metabolic pathways, whereas epigenomics, epitranscriptomics and epiproteomics explore the biochemical modifications that occur on DNA, RNA and proteins, respectively, and the way they subsequently interfere with the regulation of a vast array of cellular pathways [3,4,5].

**Figure 1:**
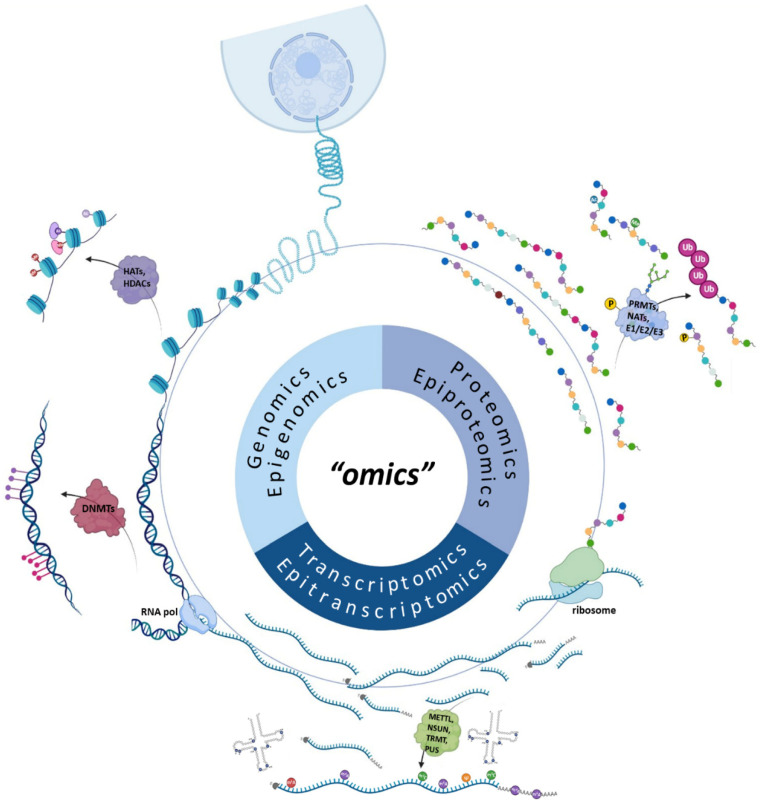
The realm of “omics”. The current sequencing approaches allow the decoding of genetic information at multiple levels. Today, DNA sequencing can be performed to decipher the nucleotide sequence of our genes as well as their epigenetic marks (genomics and epigenomics), while RNA sequencing allows the exploration of transcriptomes and the detection of RNA modifications (transcriptomics and epitranscriptomics). Finally, developing sequencing approaches that aim to investigate proteins (proteomics and epiproteomics) will lay the groundwork for a deeper understanding of cellular function and structure.

Each omics approach can shed light on just one dimension of the—otherwise exceptionally multifaceted—biological systems. However, combining data from multiple single-omics analyses and thus employing a multi-omics strategy has proven to be the key to unveil intricate biological phenomena in unprecedented detail. For example, it has been revealed that histone modifications can play a major role in the regulation of the alternative splicing process by determining the exons that will be included or skipped in the mature mRNA (Figure 1).

As a result, a combinatory implementation of diverse techniques examining not only epigenomic alterations but also their impact on gene expression is required to meticulously investigate the interrelationship between epigenome and transcriptome. Especially in clinical studies, the etiology of several pathological phenotypes usually lies in more than one omics layer, rendering the utilization of different omics technologies in a correlated manner inevitable [2,6].

Unraveling cancer etiopathogenesis has proven to be an especially challenging task since the mechanisms that drive tumor development and progression are far from simple. In fact, acquiring the hallmarks that enable the transition of cells from physiological to malignant state, such as uncontrolled proliferation, cell death evasion, bypassing growth suppressors, unlimited replicative capacity, angiogenesis induction and metastatic behavior, requires deregulation of the cellular machinery in multiple molecular levels [7]. Specifically, an astonishing amount of research has revealed a wide spectrum of defects that cooperatively promote carcinogenesis, including genomic abnormalities, epigenomic alterations and disturbance of gene transcription, as well as post-translational protein modifications. These findings suggest that the adoption of a multidimensional approach can provide a much more precise and comprehensive picture of the tumor landscape, hence serving as a powerful tool in the fields of cancer research and precision oncology [8].

Over the course of decades, the advent of high-throughput omics technologies has facilitated the thorough investigation of the molecular mechanisms that underlie tumorigenesis—sequencing undeniably being one of the main protagonists. Knowing that the complete characterization of whole genomes could be a game changer in the pursuit of answers, large-scale projects have been launched, such as The Cancer Genome Atlas (TCGA) [9] and the International Cancer Genome Consortium (ICGC) [10], which not only revolutionized our understanding on the mutational driving forces behind tumor growth, but also led biological sciences in an unprecedented era of sequencing technologies.

In fact, the need of more efficient technologies gave rise to a new generation of sequencing technologies in 2004, known as next-generation sequencing (NGS), which facilitated the rapid and cost-effective delineation of thousands of cancer genomes, thus providing a staggering amount of data on previously unexplored cancer-related genomic regions [11]. The journey however did not end there, since a few years later Third-Generation Sequencing (TGS) technologies entered the game and provided alternative sequencing approaches that overcame the limitations of NGS techniques and introduced new possibilities in the multi-omics of cancer research [12].

In the present review, we discuss the current and emerging applications of both generations of sequencing technologies, also referred to as massive parallel sequencing (MPS), in the fields of cancer genomics, transcriptomics and proteomics, as well as in the progressing realms of epi-omics. In addition, we provide a brief insight into the expanding scope of sequencing applications in personalized cancer medicine and pharmacogenomics. Finally, all the abbreviations and acronyms discussed in the current work are displayed in Table 1.

## 2. The Impact of DNA Sequencing in Cancer Genomics

Genomics constitutes an interdisciplinary field in modern biology that flourished in the last decades, due to the revolution of sequencing approaches [13]. In contrast to genetics that involves the role of individual genes, genomics encompasses the description and characterization of the total number of genes in an organism (Figure 1). Advances in MPS technologies as well as the development of novel bioinformatics and computational tools have expanded the knowledge of the diverse genomic features and enabled both the functional and structural analysis of entire genomes [14]. Furthermore, genomic-based research has facilitated the understanding of even the most complicated biological systems and has radically changed our perception of public health and precision medicine [15].

As far as cancer genomics is concerned, it constitutes a field of study that aims to decipher the basis of tumorigenesis through the detection of genetic alterations that can subsequently determine cell fate [16]. Single nucleotide substitutions, indels, large structural variants (SVs), such as chromosomal rearrangements and translocations, and copy number variations (CNVs) represent the most common somatic alterations found in cancer genomes. When accumulated through the process of cell division and/or under the influence of endogenous or exogenous factors (e.g., error-prone DNA repair mechanisms, ultraviolet light, chemical carcinogens), these variations can result in cells’ aberrant proliferation and subsequently in clonal expansion and malignancy [17]. However, not every mutation is directly implicated in cancer growth. In fact, the vast majority are considered “passenger” mutations, without serving an active role in tumor development and progression, while only a small percentage, located in “cancer genes” provides significant growth advantage to the cancer cells harboring them (Table 2). Evidently, the analysis of the complex mutational signatures in multiple samples could identify driving oncogenic mutations of various cancer types in many developmental stages, which could eventually serve as promising biomarkers and/or effective therapeutic targets [18].

The advent of NGS technologies radically changed the face of cancer DNA sequencing through the introduction of a broad range of faster, more flexible and economical high-throughput approaches. Whole-exome sequencing (WES) represents one of the most frequently used applications in cancer research, that specifically targets the coding regions of the genome (exons) to unveil pathogenic and cancer-inducing variants [30]. Despite constituting approximately 1% of the whole genome, coding regions encompass more than 85% of disease-related mutations and interestingly, they also exhibit significant mutation frequencies in a wide spectrum of cancer types—the highest ones displayed in melanoma and lung cancers [31]. WES relies on the implementation of targeted enrichment strategies, divided into hybridization-based and amplicon-based strategies, which select and enrich the whole exome prior to sequencing. In brief, hybridization-based approaches harness DNA or RNA probes, either fixed on a solid surface or biotinylated, which hybridize on the desired sequences. Accordingly, amplicon-based techniques employ target-specific primers that selectively amplify the regions that are to be sequenced [32]. Since the first human exomes of breast and colorectal cancers were characterized in 2006 [33], an extraordinary number of studies have effectively applied WES, thus shedding light on somatic mutations within coding areas that are actively involved in various malignancies (Table 2). Indicatively, new variants have been revealed in samples from patients with hereditary diffuse gastric cancer, multiple myeloma and pediatric acute myeloid leukemia [34,35,36].

Nonetheless, WES fails to identify the remaining non-coding segments of the genome, such as intergenic, intronic and untranslated regions (UTRs), promoters and regulatory elements, as well as additional functional regions and repetitive sequences. Ironically, these formerly presumed “junk” areas are capable of harboring multiple cancer driving mutations, indicating that the genomic research of cancer etiology is not limited in sequencing of the protein-coding sections only. In contrast to WES, whole-genome sequencing (WGS) can characterize the total width of the genomic landscape of an organism, providing a more overwhelming coverage and a broader perspective of cancer mutational signatures. Moreover, it is more suitable for monitoring gene fusions, CNVs and large SVs, while the absence of enrichment strategies reduces bias introduction, thus minimizing the chances of missing key cancer-related alterations due to ineffective targeted amplification or probe hybridization [37]. Major limitations, however, include the decreased sequencing depth (30–60× compared to 150–200× of WES), the higher cost and the challenging requirements for bioinformatics tools, which consequently hinder its establishment in routine clinical applications [38,39].

In addition, targeted sequencing (TS) represents the third and most versatile sequencing approach that is performed to overcome the limitations of WES and WGS by combining sensitivity and accuracy with time efficiency and reduced cost. In contrast to WGS and WES, TS focuses on characterizing targeted genomic regions of interest, which are known to incorporate cancer-related variants or variants of great clinical significance (Table 3). These regions, just like in WES, are enriched prior to sequencing via amplicon-based or hybridization-based techniques, providing over 200× sequencing depth and allowing the detection of low-frequency alterations, as well as the profiling of low-input or heavily fragmented samples [40]. Furthermore, despite the existing restrictions that currently hinder their large-scale utilization, CRISPR-based approaches have been recently combined with TS, constituting a quite promising alternative approach for enrichment that ensures high specificity, simplicity, rapid workflow and variable length of the targeted sequences [41]. Lastly, it is worth noting that, due to its flexibility, low cost and high sequencing yield, TS has emerged as a powerful diagnostic and prognostic tool in the clinical arena through the development of multiple NGS gene panels, such as the FDA-approved Oncomine™ Dx Target Test, MSK-IMPACT^®^ and FoundationOne^®^CDx. Each panel is designed to detect specific molecular alterations in a fixed number of genes, providing information on tumor classification and progression and even guiding decisions regarding the selection of targeted and patient-oriented therapeutic strategies [42,43].

**Figure 2:**
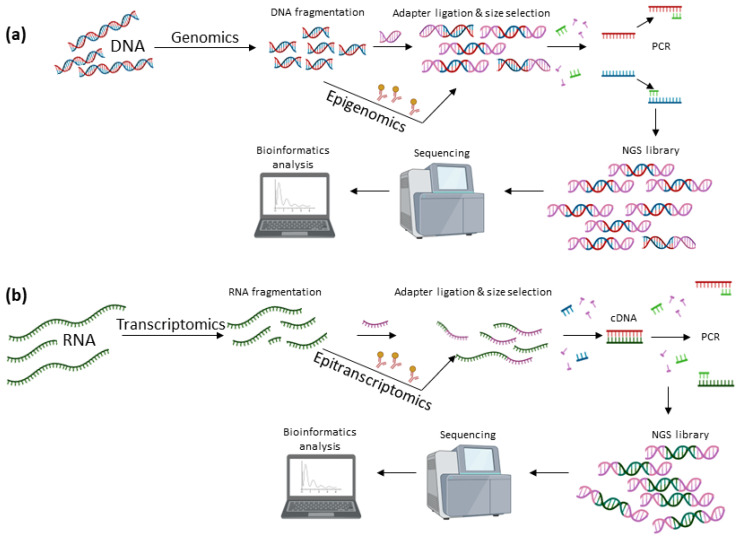
Flowchart displaying the fundamental steps of NGS workflows. (**a**) For genomic and epigenomic studies, the library preparation includes the DNA extraction and fragmentation and the ligation of the adapters on the derived DNA fragments. After the size selection, the molecules are amplified via PCR and subsequently the generated NGS library is sequenced. The final step of this process is the analysis of the obtained sequencing data using appropriate algorithms. (**b**) For transcriptomic and epitranscriptomic studies, the construction of RNA libraries is similar to the DNA library preparation workflow, but they are distinguished due to an additional reverse transcription step prior to the PCR amplification. Notably, the identification of both DNA and RNA modifications is based on the usage of specific antibodies that capture the sequence of interest.

Even though WES, WGS and TS have been emerged and gained ground through the numerous NGS platforms, the state-of-the-art TGS approaches of Pacific Biosciences^®^ (PacBio) and Oxford Nanopore Technologies Inc. (ONT) are currently taking the wheel in the field of DNA sequencing [87]. The well-established technologies of Illumina^®^ and Ion Torrent™ that remain in the frontline all these years, are mainly restricted by the short read length, which impedes the accurate characterization of larger genomic regions, such as repetitive sequences, larger insertions or deletions, CNVs and SVs. Moreover, NGS workflows include amplification steps with polymerase chain reaction (PCR), as a result incorporating biases and hindering the identification of regions rich in GC and AT bases (Figure 2).

Undoubtedly, the significantly increased read length, which usually exceeds 10 kilobases, demonstrates the most important feature of TGS technologies that can be extremely beneficial in the comprehensive characterization of genomes under normal and pathological conditions [88]. In addition, the option for PCR-free applications provided by TGS technologies enables the absolute and bias-free quantification of genomic signatures, despite the large amount of starting material that is required. However, both PacBio and ONT are characterized by increased error rate (10–15%), rendering the implementation of the more accurate short-read NGS protocols or hybrid approaches, in which long reads are corrected by NGS reads, an unavoidable part of genomic mutation studies [89]. Nevertheless, further improvements in both generations of sequencing are currently in progress and will definitely turn a new page in the genomic era of cancer research.

Last but not least, single-cell DNA sequencing (scDNA-seq) is an NGS-based approach that is rapidly gaining new ground, orientating genomics from bulk DNA sequencing of thousands or millions of cells towards the sequencing of the DNA content of individual cells [90]. Particularly in cancer research, scDNA-seq allows the genome sequencing of small or rare cell populations, such as circulating tumor cells (CTCs), as well as the identification of somatic alterations in single cells originating from different genetic lineages. Therefore, it constitutes an approach that can provide a thorough understanding of tumor evolution, metastasis and intratumoral heterogeneity, contributing to efficient cancer diagnosis, prognosis and treatment [91].

Single-cell sequencing (SCS) workflows can be summarized in four steps: isolation of single-cells from tissues, nucleic acid amplification, sequencing and bioinformatic analysis. Numerous methods have become available for whole-genome amplification (WGA) of single cells’ initial low amounts of DNA, such as DOP-PCR and LM-PCR, as well as the more efficient MDA and MLBACs techniques, which are known to introduce less amplification bias [91,92]. Finally, SCI-seq and strand-seq constitute two recent scDNA-seq approaches, which focus on the accurate CNV identification and the characterization of each homolog chromosome’s sequence respectively, while an increasing number of novel SCS strategies are constantly entering the field of DNA sequencing [93].

## 3. Epigenomics in Cancer Research

Acknowledged also as “the second dimension to the genome”, epigenome encompasses a wide repertoire of heritable molecular alterations that modulate gene expression and consequently cells’ phenotypes without however modifying the actual DNA sequence [94]. DNA methylation, histone modifications and non-coding RNAs (ncRNAs) constitute the primary epigenetic mechanisms that confer an additional layer of regulation in DNA transcription, contributing to multiple biological processes, including development, cell differentiation, cell cycle control and inactivation of X chromosome [5,95]. Aberrant patterns of DNA methylation, disturbance of post-translational modifications (PTMs) of histones and irregular expression of numerous ncRNAs have been associated with the initiation and progression of several cancer types, propelling scientific research towards the comprehensive mapping of epigenetic marks, as well as the identification of potential cancer biomarkers and promising therapeutic targets [96]. Particularly during the past decades, the unparalleled deluge of advancements in MPS technologies has revolutionized epigenomics—the genome-wide study of epigenetic codes—radically transforming our perception of the, anything but plain, human epigenetic landscape.

DNA methylation represents the most extensively explored feature of the human epigenome that is mainly defined by the methylation of the cytosine ring at its fifth carbon and thus the conversion of cytosine into the most encountered modified base in eukaryotic organisms, 5-methylcytosine (5mC) [97]. This modification primarily takes place in cytosine-phosphate-guanine (CpG) dinucleotides, accumulated in clusters known as CpG islands that exist in more than 60% of gene promoters and are also distributed all over gene regions, repetitive sequences, and additional regulatory elements, such as enhancers and insulators [98]. Hyper- and hypo-methylation of CpG islands have been associated by an astounding number of studies with gene transcriptional silencing and activation respectively, highlighting DNA methylation’s decisive contribution to the dynamic regulation of gene expression. Additionally, accumulating evidence of aberrant methylation profiles in multiple cancer types, such as breast [99], gastric [100], colorectal [101] and thyroid cancer [102], has underlined the linkage between epigenome deregulation and tumorigenesis and fueled an exponential growth of sequencing-based DNA methylation mapping techniques.

Modern sequencing approaches that are mainly focused on the accurate methylome profiling can be categorized into three groups: sodium bisulfite conversion-based, affinity enrichment-based and restriction enzyme digestion-based methods [103]. Each category employs diverse DNA pre-treatments that aim at preserving the methylation code, instead of erasing it, as in the case of PCR amplification-based sequencing approaches (Figure 2).

First and foremost, bisulfite genomic sequencing, which has been considered for decades as the gold standard for methylome characterization, relies on the pre-treatment of genomic DNA with sodium bisulfite. This leads to the deamination of unmethylated cytosines, their conversion into uracils and consequently the identification with NGS of only the unconverted, methylated cytosines’ positions [104]. In the same manner, whole-genome bisulfite sequencing (WGBS) [50], also referred to as BS-seq, methyl-seq or methylC-seq, can determine the methylation state of theoretically every single CpG site on the total width of the genome, whereas reduced representation bisulfite sequencing (RRBS) provides a much more cost-effective alternative that achieves high sequencing coverage with significantly reduced overall cost [51]. In specific, the integration of bisulfite sequencing with the use of restriction enzymes (e.g., Msp I), enriches targeted CpG-rich regions, such as promoters, which despite constituting less than 3% of the whole genome, they compose more than 80% of the total CpG content.

Lastly, due to the inability of bisulfite sequencing to discriminate between 5mC and other modified bases, like 5-hydroxymethylcytosine (5hmC), 5-formylcytosine (5fC) and 5-carboxylcytosine (5caC), many approaches have been proposed that specifically determine the positions of 5mC (oxidative bisulfite sequencing, OxBS-seq [52]), 5hmC (TET-assisted bisulfite sequencing, TAB-seq [53]), 5fC (5fC chemically assisted bisulfite sequencing, fCAB-seq [54]) and 5caC (chemical modification-assisted bisulfite sequencing, CAB-seq [55]).

On the other hand, affinity enrichment-based methods rely on proteins that target and enrich methylated sequences through less expensive and laborious workflows as compared to bisulfite sequencing. For instance, methylated DNA immunoprecipitation sequencing (MeDIP-seq) utilizes monoclonal antibodies that bind to 5mC in single-stranded DNA [56], whereas methyl-CpG-binding domain sequencing (MBD-seq) exploits MBD proteins integrated into beads to exclusively capture methylated double-stranded genomic sequences [57]. On the contrary, methylation-sensitive restriction enzyme sequencing (MRE-seq), one of the most widely used restriction enzyme digestion-based methods, makes use of multiple methyl-sensitive endonucleases that cleave double-stranded DNA at unmethylated CpGs [58]. The created fragments are then sequenced, revealing the CpG sites located at enzyme recognition sites. Moreover, several studies describe a combination of MRE-seq with MeDIP-seq or MRE-seq with bisulfite sequencing (methylation-sensitive restriction enzyme bisulfite sequencing, MREBS), which enable a more accurate genome-wide methylation status of CpGs [58].

Over the last few years, a cascade of emerging approaches has been constantly accelerating the progress of epigenetic research. First, the introduction of TGS technologies opens the way for the direct methylome profiling of native genomic DNA, without requiring amplification and chemical or enzymatic pre-treatments, thus significantly increasing the total number of DNA modifications that can be identified. Single-molecule real-time (SMRT) sequencing of PacBio^®^, for example, has the potential to detect base modifications in the DNA sequence by monitoring the perturbation of polymerase’s kinetics and the alterations in inter-pulse duration between successive base incorporations [105]. On the other hand, as methylated DNA strands translocate through the nanopores in ONT^®^ sequencing, the presence of base modifications produces distinctive electrical current deviations that can then be decoded with the use of appropriate algorithms [106]. Lastly, through the adoption of SCS approaches, such as single-cell WGBS (scWGBS) and single-cell RRBS (scRRBS), the field of human methylome analysis at the single-cell level is capturing increasingly more attention, continuously bringing us one step closer to the deeper understanding of its complexity [107].

Apart from DNA methylation, histone modifications represent another major epigenetic mark that governs chromatin accessibility, nucleosome dynamics and consequently gene transcription [108]. Post-translational modifications of specific residues, primarily located on histone tails, such as acetylation, methylation, phosphorylation, ubiquitylation, ADP-ribosylation, sumoylation and deamination, can interfere with chromatin conformation and the recruitment of several protein complexes, influencing as a result a wide range of cellular processes such as transcription, DNA replication and DNA repair. For instance, hyperacetylation is correlated with a more “open” and accessible to protein factors chromatin structure, thus leading to activation of gene transcription, whereas deacetylation is associated with a more compacted chromatin conformation and hence gene silencing [109]. Interestingly, numerous mutations in histone-modifying enzymes have already been identified and linked with disruption of modification patterns, irregular transcription of tumor-associated genes and cancer development, thus prompting the development of sequencing-based techniques aiming at the genome-wide decryption of histone marks [110].

Chromatin immunoprecipitation followed by sequencing (ChIP-seq) constitutes the primary method for identifying histone modification positions, as well as chromatin-associated proteins binding sites. In brief, this method involves DNA fragmentation through micrococcal nuclease (MNase) digestion, binding of antibodies on specific histone marks, purification and lastly sequencing of the enriched DNA fragments [63]. Alternatively, to profile transcription factors’ binding sites, proteins initially become crosslinked to the DNA sequence, followed by chromatin fragmentation, immunoprecipitation and sequencing. ChIP-seq has also been adapted to decode epigenetic marks at single-cell resolution through the establishment of single-cell ChIP-seq (scChIP-seq) approaches [111]. Droplet-based single-cell ChIP-seq (Drop-ChIP), for instance, relies on the use of microfluidics devices, which can process individual cells captured within aqueous drops [112], whereas single-cell simultaneous indexing and tagmentation-based ChIP-seq (sc-itChIPseq) represents a more low-priced and widespread approach employing Tn5 transposase [113]. Furthermore, due to the biases introduced in multiple steps of ChIP-seq workflows, numerous enzyme-tethering alternative methodologies have been developed, such as Cleavage Under Targets and Release Using Nuclease (CUT&RUN) [64] and Cleavage Under Targets and Tagmentation (CUT&Tag) [65], known to provide high-resolution and high-throughput data on epigenome profiling (Table 3). Ultimately, chromatin accessibility, nucleosome positioning, as well as the formation of higher-order chromatin structures can be shaped by the presence of histone modifications and determine protein binding, interaction dynamics and eventually gene expression. These epigenetic features can be accessed via numerous techniques, such as DNase I hypersensitive sites sequencing (DNase-seq) [66], formaldehyde-assisted isolation of regulatory elements with sequencing (FAIRE-seq) and assay for transposase-accessible chromatin using sequencing (ATAC-seq) for chromatin accessibility [67], MNase digestion of chromatin followed by sequencing (MNase-seq) for nucleosome positioning [68] and Hi-C [69] and chromatin interaction analysis with paired-end tag (ChIA-PET) for studying chromatin architecture (Table 3) [70].

Lastly, piling evidence have highlighted the critical role of ncRNAs in the regulation of the epigenetic landscape of cells [114]. For example, microRNAs (miRNAs) can repress gene expression as part of the RNA-induced silencing complex (RISC) by binding on the 3′UTR of the messenger RNA (mRNA), as well as target and regulate the expression of epigenetic modifiers (e.g., DNA methyltransferases, histone deacetylases), thus playing part in the sculpting of methylome and histone modification patterns [115]. Small interfering RNAs (siRNAs) can also induce gene silencing through the RNA interference (RNAi), whereas long non-coding RNAs (lncRNAs) can serve as molecular signals, guides of chromatin modifiers to specific genomic sequences, scaffolds for the formation of protein complexes, as well as decoys interacting with transcription factors and promoting or repressing gene expression [116].

In the past decade, major advances in high-throughput sequencing approaches have facilitated the detailed study of ncRNAs. First and foremost, RNA sequencing (RNA-seq) is a versatile tool that has radically transformed transcriptome analysis and has also been adapted for the accurate identification and quantification of lncRNAs (long RNA-seq) and small ncRNAs (small RNA-seq), as well as for the precise assessment of specifically the expression levels of miRNAs (miRNA-seq) [117,118]. On the other hand, RNA immunoprecipitation sequencing (RIP-seq) can be efficiently employed to examine protein–ncRNAs interactions, utilizing specific antibodies against targeted RNA binding proteins.

By immunoprecipitating specific RNA-protein complexes, isolating the RNA and sequencing, researchers can thoroughly investigate the protein-ncRNAs binding sites of interest, thus broadening our knowledge on the complexity of their biological functions [72]. High-throughput sequencing of RNA isolated by crosslinking immunoprecipitation (HITS-CLIP) is another technique that permits the comprehensive study of RNA–protein interactions on a genome-wide scale, being particularly valuable for the identification of Argonaute–miRNAs and Argonaute–mRNAs interplays in RISC [73]. A more popular and efficient alternative approach, photoactivable-ribonucleoside-enhanced-CLIP (PAR-CLIP), relies on the use of photoreactive ribonucleosides for crosslinking enhancement rather than UV-irradiation [119]. In addition, cross-linking ligation and sequencing of hybrids (CLASH) and ligation of interacting RNA followed by high-throughput sequencing (LIGR-seq) are suitable for the characterization of RNA–RNA interaction networks, having already highlighted the presence of a wide array of miRNA–mRNA binding sites [75,76].

As far as ncRNAs–chromatin interactions are concerned, chromatin isolation by RNA purification (ChIRP) and capture hybridization analysis of RNA targets (CHART) can examine the chromatin interaction sites of a single lncRNA at a time [77,78], while approaches like global RNA interactions with DNA by deep sequencing (GRID-seq) and chromatin-associated RNA sequencing (ChAR-seq) are capable of mapping the totality of RNA-chromatin interactions present within cells [79,80]. Furthermore, several methodologies, focusing on the high-resolution characterization of RNA structures, have also become available, including the selective 2′-hydroxyl acylation analyzed by primer extension sequencing (SHAPE-seq) [81], selective 2′-hydroxyl acylation analyzed by primer extension and mutational profiling (SHAPE-MaP) [82] and in vivo click selective 2′-hydroxyl acylation and profiling experiment (icSHAPE) [83]. Finally, it is worth mentioning that nanopore sequencing offers approaches suitable for the precise characterization of small RNAs like miRNAs, significantly expanding our toolkit in epigenome exploration.

## 4. The Advent of RNA Sequencing for Transcriptome Profiling

Transcriptome signifies the entire set of RNA molecules within cells, encompassing their transcription and expression levels as well as their structure, molecular functions and cellular locations [120]. The transient nature of RNA’s life renders transcriptome a highly dynamic entity which captures a snapshot in time of the RNA molecules that are being transcribed. Accordingly, RNAs are subjected to multiple biological processes that increase transcriptomic complexity and may differ under physiological or pathological conditions and hence cancer. The study of the transcriptome, referred to as transcriptomics, involves the characterization of the all the aforementioned RNA components, as well as the identification of alternative splicing events and polyadenylation sites and the detection of ncRNAs, such as microRNAs and lncRNAs, and fusion transcripts [120].

Modern transcriptomics harness high-throughput sequencing approaches that enable the efficient sequencing of the existing RNA molecules in a sample and the accurate mapping of the characterized sequences to a reference genome [121]. The last decade multiple RNA-sequencing (RNA-seq) methods have been developed for transcriptome analysis and resolution at the nucleotide level. Of note, all NGS platforms as well as the third-generation sequencers of PacBio, that are based on sequencing-by-synthesis technology, share a standard workflow that involves RNA isolation, mRNA enrichment, cDNA synthesis and RNA-seq library preparation [71,122] (Figure 2). On the contrary, the cutting-edge technology of nanopore sequencing not only allows sequencing of cDNA libraries, but is also able to perform direct sequencing of RNA molecules for the first time (Direct RNA-seq) [123]. In this approach, full-length RNAs can be directly sequenced, avoiding the reverse transcription and the amplification bias and as a result enabling their absolute quantification in human cells. Even though there are still a few limitations, this method shows great promise in the following years to completely transform not only transcriptome analysis, but also the field of epiranscriptomics. Moreover, as with WGS and WES, whole-transcriptome sequencing (WTS) enables the identification of both coding and ncRNAs and unveils known and novel features of the transcriptome, whereas, in the same manner as TS in genomics, the highly accurate application of targeted RNA-Seq focuses on specific transcripts of interest that are firstly selected by amplicon-based approaches or enrichment of the target and are then sequenced.

Both RNA-seq techniques facilitate the study of transcriptome in terms of fusion transcripts, alternative spliced mRNAs and ncRNAs. In fact, fusion transcripts, resulting from gene fusion events, are present in ~20% of tumors, while alternative splicing can generate various mRNAs that are associated with multiple cancerous situations [124]. RNA-Seq methods have the capacity to successfully identify these chimeric and/or alternative spliced RNAs, many of which are involved in cancer progression and development and hence they represent putative biomarkers for cancer diagnosis and prognosis [71]. For instance, the mRNAs produced by the translocation of *BCR* and *ABL1* genes represent typical examples of oncogenic fusion transcripts that characterize chronic myeloid leukemia. Several studies support that *BCR-ABL1* fusion generates multiple fusion transcripts, which encode oncoprotein isoforms that subsequently participate in signaling pathways promoting tyrosine kinase activity [125]. Regarding cancer research, WTS represents the optimal approach for detecting novel fusions, whereas targeted transcript panels aim to identify alternative spliced mRNAs candidates for cancer biomarkers. Lastly, although mRNAs are a matter of great interest, MPS also permits small RNA-seq that allows the profiling of additional RNA species, including ncRNAs, which have been found to interfere with complex cellular networks and hence act as tumor promoters and/or suppressors [71,120].

The innovative technologies of MPS have already established RNA-seq as the preferred strategy for analyzing the relative abundance of mRNA transcripts. The newly introduced digital gene expression sequencing (DGE-Seq) represents a high-throughput sequencing approach that identifies statistically significant differences in RNA abundance through the construction and sequencing of cDNA libraries [126]. Notably, DGE analysis is of foremost interest to both basic and clinical research since it provides considerable insight into the transcriptional mechanisms and biological functions that contribute to phenotypic differences leading to pathogenesis and tumorigenesis.

Ultimately, the emerging single-cell RNA-seq (scRNA-seq) technology has revolutionized our understanding on cancer dynamics by defining the transcriptome of individual cells and studying cell heterogeneity [127]. More specifically, scRNA-seq allows the investigation of intercellular transcriptional variability and provides in-depth analysis of the mechanisms that are involved in cancer progression and metastasis, thus being considered the gold standard for cancer transcriptomics [127,128]. To date, several scRNA-seq strategies are available, among which RAGE-Seq and SMART-Seq are the most popular [129,130].

Briefly, RAGE-Seq is a high-throughput method that utilizes nanopore’s full-length sequencing reads and has been used for tracking transcriptome profiles of lymphocytes to study immune cells and eventually target different cancer stages. On the contrary, SMART-seq requires the construction of standard Illumina sequencing libraries and it has been used for profiling transcriptome in rare cells such as melanoma tumor cells [129]. Evidently, the goal of these approaches is to characterize outliers within a cellular population and expand our knowledge on cancer biomarkers, drug response and treatment. Undoubtedly, the expanding scope of pioneering applications provided by sequencing platforms is driving scientific research on the verge of a new era in transcriptomics, which will inevitably impact our understanding and the therapeutic strategies employed in cancer research.

## 5. The Golden Era of Epitranscriptomics in Cancer Research

Since the RNA complexity came into focus, multiple methodological strategies and technological breakthroughs uncovered the hidden aspects of the transcriptome, hence repeatedly enhancing our efforts to decipher the epitranscriptional landscape of the human cells [131,132]. Epitranscriptome involves the study of different types of RNA modifications that generally regulate gene expression by affecting RNA stability, translation, RNA degradation and miRNA binding. Briefly, more than 170 different types of post-transcriptional modifications have been described to date to decorate multiple types of RNAs, among which N^6^-methyladenosine (m^6^A), 5-methylcytosine (m^5^C), N^1^-methyladenosine (m^1^A), pseudouridine (Ψ) and adenosine to inosine (A-to-I) are found to be the most abundant [131,133].

The complex features of RNA modifications and their dynamic interacting networks operating at post-transcriptional levels are nowadays at the heart of modern cancer research. Multiple studies point out the involvement of RNA modifications in signaling pathways leading to tumorigenesis [131]. To begin with, the most extensively studied m^6^A methylation pathway has been reported to affect various cancer types. In brief, the methyltransferase-like METTL3/METTL14 complex, known as “m^6^A writers”, is responsible for the deposition of m^6^A in mRNAs, ncRNAs and rRNAs, whereas alkB homologue 5 (ALKBH5) and fat mass and obesity-associated protein (FTO) belong to ‘m^6^A erasers’ that demethylate the RNA [134,135]. “Reader” proteins of the YTH domain-containing family recognize the m^6^A sites thus mediate translation and degradation. Interestingly, METTL3 promotes tumor progression by regulating the translation of oncoproteins in different human cancers such as liver, lung and endometrial cancer. For instance, in lung cancer the oncogenic role of METTL3 promotes the expression of EGFR, whereas in endometrial cancer, it activates the AKT pathway [136,137].

In the same manner, NSUN1-7 “writers”, members of the NSUN family, are involved in the methylation of cytosine at position 5, while members TET family erase the m^5^C sites [134]. As for their recognition, the RNA m^5^C binding proteins, ALYREF and YBX1, are considered to read cytosine modifications [135]. Accordingly, several studies have already elucidated the connection between m^5^C and human malignancies. For example, in human urothelial carcinoma of the bladder, NSUN2 and YBX1 are responsible for the stabilization of the oncogenic mRNAs through the deposition of m^5^C sites and the upregulation of the hypermethylated sites, respectively. On the contrary, the proliferation role of NSUN1 is upregulated in lung and prostate cancer [138,139]. Similarly, m^1^A is found in both nuclear and mitochondrial mRNAs as well as in tRNAs and rRNAs, being mainly deposited by tRNA methyltransferase 6 non-catalytic subunit (TRMT6)/61A, TRMT61B and TRMT10C. On the other hand, ALKBH1 and ALKBH3 mediate the demethylation of m^1^A, whereas the YTH domain-containing protein family detects the methylated sites. Recent studies support the implication of m^1^A in human cancers by modulating vital signaling pathways, such as mTOR and AKT. In pancreatic adenocarcinoma, the downregulation of ALKBH1 affects m^1^A levels and has been related to the poor prognosis of patients, while in breast and ovarian cancer, demethylation of m^1^A by ALKBH3 regulates the mRNA stability of the survival factor CSF-1 hence extending its lifespan [140].

Pseudouridylation, the most abundant modification across the RNAs in human cells, is involved in ribosome assembly, tRNA structure and stability and mRNA splicing [141]. A total 14 different pseudouridylases of the pseudouridine synthase (PUS) family target different types of RNA and modify the Ψ sites [142]. For example, PUS1 is involved in the modification of snRNAs, tRNAs and mRNAs, PUS7 is linked to tRNA modifications, whereas PUS10 relates to tRNA pseudouridylation and miRNA processing. As far as cancer is concerned, PUS1 modulates interactions between SRA1 and estrogen receptor in breast cancer, PUS7 has a putative role in ovarian cancer diagnosis, while in prostate cells, PUS10 regulates TNF-related apoptosis-inducing ligand (TRAIL) [143,144,145]. Accordingly, A to I conversion, which is found in tRNAs, primary mRNAs and miRNAs, is mediated by ADAR family by deaminating adenosine and alters RNAs secondary structures, splicing regulation and miRNA specificity [146]. It should be noted that in breast cancer, ADAR-mediated RNA editing promotes tumor progression, whereas in hepatocellular carcinoma, ADAR1 contributes to increased cell proliferation by editing *AZIN1* mRNA [147].

**Figure 3:**
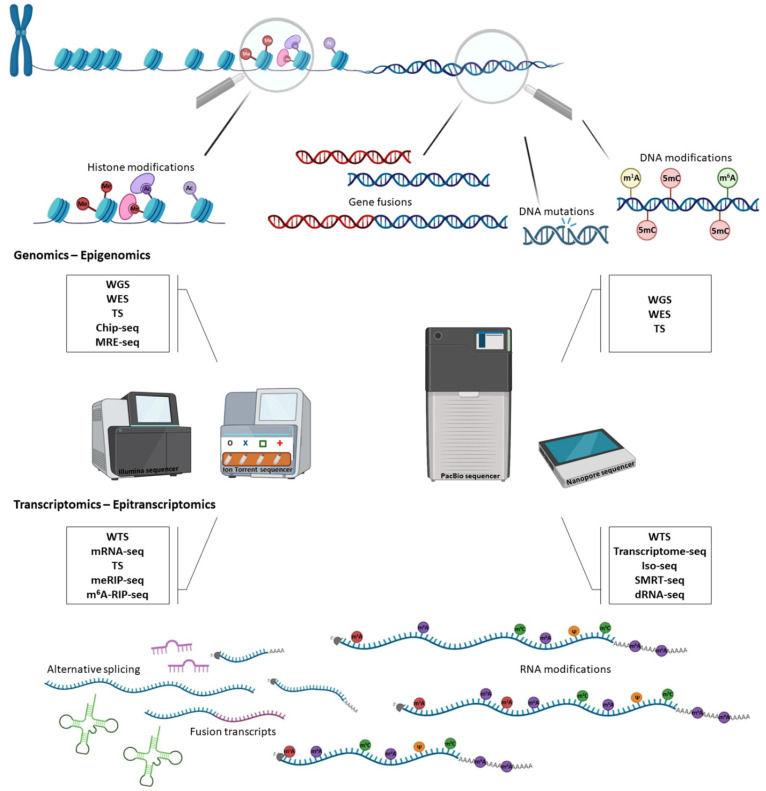
Different levels of genetic information and the related applications of MPS technology. DNA sequencing applications aim to detect DNA modifications, mutations, gene fusions and histone modifications, whereas sequencing-based RNA studies include the identification of alternative splicing events, transcript fusions and RNA modifications.

Although in the past the study of epitranscriptome was based on mass spectrometry [148,149], RNA-seq approaches and their applications have already gained ground enabling a better study of RNA modifications (Table 4). Both NGS and TGS have already provided important insights into the characterization of epitranscriptomic marks and allowed transcriptome-wide mapping of RNA modifications (Figure 3) [150]. Notably, a growing number of detection methods are being developed for mapping RNA modifications and most of them are based on several different strategies [132]. More precisely, the first approach utilizes reverse transcription coupled to NGS and is based on detecting mismatched dNTPs that are incorporated during cDNA synthesis on account of the presence of modified nucleotides. On behalf of this strategy, DART-seq and m^1^A-IP-seq/m^1^Aquant-seq generate modification-dependent mutational signatures for the identification of m^6^A and m^1^A sites, respectively [151,152].

Antibody-based mapping approaches are the most common sequencing-based strategies for studying RNA modifications (Figure 2). The method combines highly specific antibodies that enrich modification sites, and high-throughput sequencing for the detection of the immunoprecipitated RNA fragment [153]. For instance, the widely used m^6^A-seq and MeRIP-seq protocols, have been developed to capture m^6^A sites in RRACH consensus motifs [154]. Accordingly, m^5^C-RIP-seq and Aza-IP use appropriate antibodies to capture m^5^C sites, thus enabling the recognition of these sites into the RNA [153,155]. On the contrary, several modifications can be detected based on their differential chemical reactivity, such as Ψ that can be identified through Pseudo-seq, Ψ-seq and PSI-seq approaches. The principle of Pseudo-seq relies on the differential chemical reactivity of Ψ when treated with Cyclohexyl-Methylmorpholino-Carbodiimide (CMC) [156]. Ψ-CMC induces reverse transcription arrest one nucleotide downstream to the Ψ site and enables its detection through NGS. Additional strategies, such as PAR-CLIP, aim to improve mapping accuracy and achieve single-nucleotide resolution [74].

**Table 4:** Transcriptome and epitranscritome profiling through applications based on RNA-seq.

Transcriptomics/ Epitranscriptomics	Technology	Application	References
Fusion transcripts	NGS	WTS, TS	[157,158,159]
	TGS	SMRT-seq, Direct RNA-seq	[160]
Alternative splicing	NGS	RNA-seq, targeted RNA sequencing	[161,162]
	TGS	Direct RNA-seq, WTS	[160,163]
mRNA polydadenylation	NGS	3′ enriched RNA seq, 3′ mRNA seq, PAT-seq, Poly(A) ClickSeq	[164,165,166,167]
	TGS	Full length mRNA seq, FLAM-seq, Long read cDNA-seq	[168,169]
ncRNAs/lncRNAs	NGS	scRNA-seq, WTS, WES, AQRNA-seq, ncPRo-seq, miRNA-seq	[170,171,172,173]
	TGS	Nanopore-induced phase-shift sequencing (NIPSS)	[86]
m^6^A	NGS	Transcriptome-wide m^6^A seq, m^6^A-RIP seq, m^6^A-REF seq	[174,175]
	TGS	Direct RNA-seq	[176]
m^5^C	NGS	RNA-BisSeq, RIP-seq, MeRIP-seq, m5C-RIP seq, AZA-IP seq	[153,154,155,177]
	TGS	Direct RNA-seq	[178]
Ψ	NGS	Pseudo-seq	[156]
	TGS	Direct RNA-seq	[179]
m^1^A	NGS	ARM-seq, m^1^A-quant seq, m^1^A-seq	[180,181,182]

Although NGS approaches have been established in the scientific community for studying RNA modifications, the advent of TGS platforms have changed our perspectives in epitranscriptomics (Table 4). The amplification-free single-molecule sequencing approaches of TGS, which include PacBio’s SMRT sequencing and ONT’s direct RNA sequencing, rely on the detection of the kinetic signatures on the nucleotides [183]. In SMRT, detection of RNA modifications is succeeded by coupling RT with SMRT. The DNA polymerase can “read” the dynamic signals of RT and discriminates the modified from the unmodified nucleotides [184]. As for the state-of-the-art nanopore direct RNA sequencing, it enables the direct sequencing of full-length native RNAs, without the need for reverse transcription or amplification. RNA modifications through nanopore sequencing can be identified by monitoring ionic current changes [185,186]. Ultimately, rather than the PCR-independent strategy, the major advantage of TGS is the single-molecule analysis that will definitely provide the blueprints of epitranscriptome.

## 6. The Road Ahead in Proteomics and Epiproteomics

The large-scale study of proteomes involves the identification and quantification of proteins, the building blocks of life, which are expressed in a cell, tissue or an individual and is widely known as proteomics [187]. Besides protein expression profiling, the field of proteomics also comprises the structural and functional determination of proteins, whereas protein isoforms and post-transcriptional modifications, including histone acetylation, phosphorylation and ubiquitination, constitute the epiproteomic signatures of cells [188]. Although advances in sequencing technologies have resulted in an explosion of the available amount of omics data, proteomic studies have yet to attain the power of both genome and transcriptome research [189,190].

Heretofore, Edman degradation and mass spectrometry were the benchmark for protein sequencing. However, recent trends in proteomics have opened up the fascinating possibility of sequencing thousands to millions of peptide molecules in parallel [191]. In the following years, innovative methods, such as fluorosequencing and nanopore sequencing, will undoubtedly revolutionize proteomic studies enabling the accurate identification of low abundance protein isoforms and their post-translational modifications [192]. The method of protein fluorosequencing has its roots in next-generation sequencing, since it is based on labeling the amino acid residues with different fluorophores, immobilizing the C-terminus of the peptides on a glass coverslip and detecting the fluorescence intensity by using Edman degradation for the sequential cleavage of the N-terminal amino acid residue [193]. On the contrary, the prospect of protein sequencing through nanopore technology relies on the disturbance of the electric current when amino acid residues pass through the channel of the nanopore. Protein samples cause characteristic alterations in the density of the ionic current flowing through the membrane of the nanopore, thus any amino acid sequence can be identified [194]. Furthermore, it should be mentioned that nanopore protein sequencing has the potential to detect post-transcriptional modifications, hence paving the way to the advancing field of epiproteomics research.

Proteomics-based studies focus on protein profile analysis, which is the key to understand the dynamic nature of cells that underlies the mechanisms of tumor growth and metastasis. The field of proteomics has already contributed to the identification of novel biomarkers and potential therapeutic targets, gaining important ground in cancer research [195]. For instance, ever since the correlation between tyrosine kinase signaling and tumor growth was brought to light, multiple kinase inhibitors were developed and successfully applied to treating several cancer types, such as chronic myeloid leukemia, anaplastic lymphoma, breast and lung cancer. Finally, the ultimate goal of cancer proteomics is to adapt MPS technologies for routine use in clinical laboratories for identifying molecular characteristics to diagnose cancer or determine a patient’s prognosis [190]. Without doubt, deciphering the proteomic and epiproteomic portrait is the next frontier of cancer research to achieve multi-omics studies across tumor types and succeed in the accurate diagnostic and/or prognostic classification of disease states, as well as in evaluating drug dose and drug effect [196].

## 7. Pharmacogenomics in Medical Oncology

**Figure 4:**
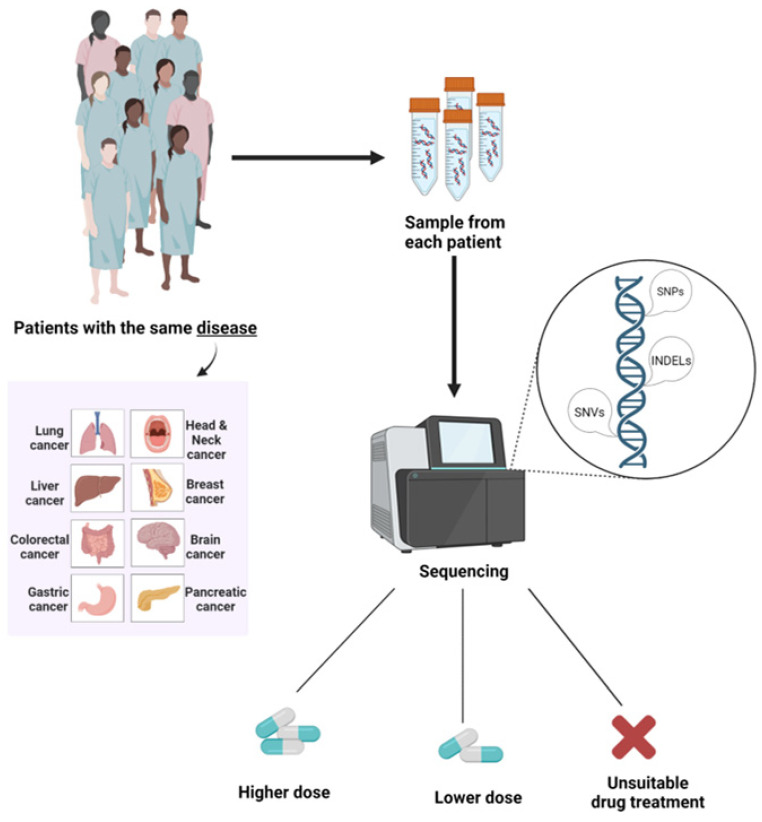
Pharmacogenomics is based on the sample collection from patients dealing with the same disease, the extraction of nucleic acids from each sample and the subsequent sequencing of them, following the appropriate workflow. After data analysis, the unique genetic profile of each patient is used as a reference point for the choice of the optimal drug treatment each one should receive.

When the first chemotherapeutic drug was discovered, no one could imagine that a drug treatment would benefit only a group of patients dealing with the corresponding disease and not all of them. Moreover, that is what personalized medicine stands for. Therefore, the question is whether the different responses to a specific treatment come from the distinctiveness of our DNA (Figure 4). According to previous studies that focus on personalized healthcare, the efficacy and toxicity of a drug treatment is significantly influenced by the genomic variation of each person, thus focusing on each patient individually is the key to a successful treatment.

Pharmacogenomics aims to study these genomic variations and decipher how genetic alterations influence drug effectiveness and toxicity, in order to develop a personalized and targeted therapy with optimized efficacy and reduced toxic effects [197]. For this purpose, studies conducted in the framework of personalized medicine have shed light in the way genetic variation across individuals affects a drug’s pharmacokinetics and pharmacodynamics. The first step to the introduction of personalized medicine is the determination of the association between a certain genotype and a drug-induced phenotype [198]. If these associations are reproducible and have great effect size, clinical use of such information can be implemented for the benefit of patients and lead to the development of personalized medical treatments.

Especially in cancer, driven somatic mutations as well as genetic alterations play an important role in treatment choice. For example, patients diagnosed with colorectal cancer display significantly prolonged survival after treatment with cetuximab, a chemotherapeutic drug that targets the EGF receptor [199]. However, in cases of colorectal cancer where EGFR is not overexpressed, cetuximab would not be a possible treatment choice. When dealing with cancer patients, pharmacogenomics plays a critical role, since tumors are characterized by a great heterogeneity. Every case should be treated uniquely to achieve a better quality of a patient’s life and prolong their survival. Based on the aforementioned, it becomes clear that pharmacogenomics represents a field of biological sciences with great impact in public health.

The introduction of NGS and TGS promoted the development of personalized medicine, giving oncologists the ability to deeply understand every patient’s disease and its unique genetic features. Especially NGS, which has been used in laboratory infrastructures for a longer time compared to TGS technologies, allowed us to identify mutations that are strongly linked to tumorigenesis and cancer progression, and laid the groundwork to predict how a cancer patient will respond to a particular treatment, based on their genetic fingerprint. NGS gave the opportunity for screening a broad panel of genes related to a specific cancer type in every patient, allowing not only the detection of the most common alterations that are known, but also the identification of rare mutations that occur in less than 1% of the patients, thus providing useful information regarding drug intolerance, efficacy and toxicity. With the examination of tissue samples from biopsies, CTCs and circulating cell-free tumor DNA (ctDNA) via NGS approaches is able to obtain extensive genetic information, in order to assess intertumoral heterogeneity and decide the optimal treatment for each patient, based on the findings [200,201].

As for the newly introduced long-read technologies, Nanopore has already entered the “precision oncology” era, for the characterization of tumor genetic features in cancer patients’ management. Specifically, Nanopore sequencing has been used for the analysis of the copy number variations from cell-free DNA in lung cancer patients [198], currently for research purposes only, but exploiting the full potential of Nanopore’s establishment in both research and clinical use is needed. Taking into consideration the aforementioned, it is easy to understand the significance of NGS and TGS in the evolution of personalized oncology, an approach that has opened new horizons in patients’ treatment and drug selection.

## 8. Conclusions

The advent of high-throughput sequencing technologies has uncovered an incredible amount of biological information, holistically altering our perception about genomics, transcriptomics and proteomics, thus leading to their establishment in life science. Nowadays, multi-omics has opened new vistas for developments in modern cancer research aiming to translate omics into clinical practice for improving both diagnosis and treatment. In fact, numerous novel technologies have already improved the field of oncology by employing omics as a new clinical method for early diagnosis, prognosis, treatment and control of cancer. The implementation of sequencing-based omics techniques covers almost every aspect of cancer biomedical research, such as identification of novel biomarkers, drug discovery and personalized medicine, which will improve health outcomes. Ultimately, further advances and future studies in the omics universe will undoubtedly peek into the innermost secrets of life.

## Figures and Tables

**Table 1:** List of abbreviations and acronyms.

Abbreviation	Definition
5caC	5-carboxylcytosine
5fC	5-formylcytosine
5hmC	5-hydroxymethylcytosine
5mC	5-methylcytosine
ATAC-seq	Assay for transposase-accessible chromatin using sequencing
A-to-I	Adenosine to inosine
CAB-seq	Chemical modification-assisted bisulfite sequencing
ChAR-seq	Chromatin-associated RNA sequencing
CHART	Capture hybridization analysis of RNA targets
ChIA-PET	Chromatin interaction analysis with paired-end tag
ChIP-seq	Chromatin immunoprecipitation followed by sequencing
ChIRP	Chromatin isolation by RNA purification
CLASH	Cross-linking ligation and sequencing of hybrids
CMC	Cyclohexyl-Methylmorpholino-Carbodiimide
CNVs	Copy number variations
CpG	Cytosine-phosphate-guanine
CTCs	Circulating tumor cells
ctDNA	Cell-free tumor DNA
CUT&RUN	Cleavage Under Targets and Release Using Nuclease
CUT&Tag	Cleavage Under Targets and Tagmentation
DGE-Seq	Digital gene expression sequencing
DNase-seq	DNase I hypersensitive sites sequencing
Drop-ChIP	Droplet-based single-cell ChIP-seq
FAIRE-seq	Formaldehyde-assisted isolation of regulatory elements with sequencing
fCAB-seq	5fC chemically assisted bisulfite sequencing
GRID-seq	Global RNA interactions with DNA by deep sequencing
HITS-CLIP	High-throughput sequencing of RNA isolated by crosslinking immunoprecipitation
ICGC	International Cancer Genome Consortium
icSHAPE	In vivo click selective 2′-hydroxyl acylation and profiling experiment
LIGR-seq	Ligation of interacting RNA followed by high-throughput sequencing
m1A	N1-methyladenosine
m5C	5-methylcytosine
m6A	N6-methyladenosine
MBD-seq	Methyl-CpG-binding domain sequencing
MeDIP-seq	Methylated DNA immunoprecipitation sequencing
MNase	Micrococcal nuclease
MNase-seq	MNase digestion of chromatin followed by sequencing
MPS	Massive parallel sequencing
MREBS	Methylation-sensitive restriction enzyme bisulfite sequencing
MRE-seq	Methylation-sensitive restriction enzyme sequencing
NGS	Next-generation sequencing
ONT	Oxford Nanopore Technologies
OxBS-seq	Oxidative bisulfite sequencing
PacBio	Pacific Biosciences
PAR-CLIP	Photoactivable-ribonucleoside-enhanced-CLIP
PCR	Polymerase chain reaction
PTMs	Post-translational modifications
PUS	Pseudouridine synthase
RIP-seq	RNA immunoprecipitation sequencing
RISC	RNA-induced silencing complex
RNAi	RNA interference
RRBS	Reduced representation bisulfite sequencing
scChIP-seq	Single-cell ChIP-seq
scDNA-seq	Single-cell DNA sequencing
scitChIPseq	Single-cell simultaneous indexing and tagmentation-based ChIP-seq
scRNA-seq	Single-cell RNA sequencing
scRRBS	Single-cell RRBS
SCS	Single-cell sequencing
scWGBS	Single-cell WGBS
SHAPE-MaP	Selective 2′-hydroxyl acylation analyzed by primer extension and mutational profiling
SHAPE-seq	Selective 2′-hydroxyl acylation analyzed by primer extension sequencing
siRNAs	Small interfering RNAs
SMRT	Single-molecule real-time
SVs	Structural variants
TAB-seq	TET-assisted bisulfite sequencing
TCGA	The Cancer Genome Atlas
TGS	Third-generation sequencing
TRAIL	TNF-related apoptosis-inducing ligand
TS	Targeted sequencing
UTRs	Untranslated regions
WES	Whole-exome sequencing
WGA	Whole-genome amplification
WGBS	Whole-genome bisulfite sequencing
WGS	Whole-genome sequencing
WTS	Whole-transcriptome sequencing
Ψ	Pseudouridine

**Table 2:** High-throughput sequencing applications for the investigation of specific genomic, epigenomic and transcriptomic events in cancer research.

Gene	“omics”	Cancer Type	Technology	Application	Reference
*MYB*	Genomics/ chromosomal rearrangement	Low grade glioma	NGS	WGS	[19]
*ACTB-FOSB*	Genomics/ gene fusion	Pseudomyogenic hemangioendothelioma	NGS	Targeted RNA-seq	[20]
*KMT2A-MLTT2/4*	Genomics/ gene fusion	Acute myeloid leukemia	TGS	Nanopore sequencing	[21]
*CDKN2A*	Genomics/ mutation	Hepatocellular carcinoma	NGS	WES	[22]
*IRF-4*	Epigenomics/ DNA methylation	Lymphoma	NGS	WGBS	[23]
*KRT19*	Epigenomics/ DNA methylation	Breast cancer	TGS	Nanopore sequencing	[24]
*DBC1*	Epigenomics/ histone modification	Colorectal cancer	NGS	ChIP-seq RNA-seq	[25]
*BCR-ABL1*	Transcriptomics/ fusion transcripts	Chronic myelogenous leukaemia	NGS	Targeted RNA-seq	[26,27]
*MLH1*	Transcriptomics/ alternative splicing	Colorectal cancer	TGS	Long read RNA-seq	[28]
*CSTF2*	Transcriptomics/ alternative polyadenylation	Non-small cell lung cancer	NGS	IVT-SAPAS	[29]

**Table 3:** Genomics/epigenomics applications that have been established through MPS approaches.

Genomics/Epigenomics	Technology	Application	References
DNA mutations	NGS	WES, WGS, TS	[44,45]
Larger SVs, CNVs, gene fusions	NGS		[46,47,48]
	TGS		[21,49]
DNA methylation	NGS	WGBS	[50]
		RRBS	[51]
		OxBS-seq	[52]
		TAB-seq	[53]
		fCAB-seq	[54]
		CAB-seq	[55]
		MeDIP-seq	[56]
		MBD-seq	[57]
		MRE-seq	[58]
	TGS	SMRT sequencing	[59,60]
		Nanopore sequencing	[61,62]
Histone modifications	NGS	ChIP-seq	[63]
		CUT&RUN	[64]
		CUT&Tag	[65]
Chromatin accessibility	NGS	DNase-seq	[66]
		FAIRE-seq	[67]
		ATAC-seq	[67]
Nucleosome positioning	NGS	MNase-seq	[68]
3D genome structure	NGS	Hi-C	[69]
		ChIA-PET	[70]
ncRNAs	NGS	RNA-seq	[71]
		RIP-seq	[72]
		HITS-CLIP	[73]
		PAR-CLIP	[74]
		CLASH	[75]
		LIGR-seq	[76]
		ChIRP	[77]
		CHART	[78]
		GRID-seq	[79]
		ChAR-seq	[80]
		SHAPE-seq	[81]
		SHAPE-MaP	[82]
		icSHAPE	[83]
	TGS	Nanopore sequencing	[84,85,86]

## Data Availability

No new data were created or analyzed in this study. Data sharing is not applicable to this article.

## References

[1] Yadav S.P. (2007). The wholeness in suffix -omics, -omes, and the word om. J. Biomol. Tech..

[2] Olivier M., Asmis R., Hawkins G.A., Howard T.D., Cox L.A. (2019). The need for multi-omics biomarker signatures in precision medicine. Int. J. Mol. Sci..

[3] Manzoni C., Kia D.A., Vandrovcova J., Hardy J., Wood N.W., Lewis P.A., Ferrari R. (2018). Genome, transcriptome and proteome: The rise of omics data and their integration in biomedical sciences. Brief. Bioinform..

[4] Song H., Liu D., Dong S., Zeng L., Wu Z., Zhao P., Zhang L., Chen Z.S., Zou C. (2020). Epitranscriptomics and epiproteomics in cancer drug resistance: Therapeutic implications. Signal Transduct. Target. Ther..

[5] Wang K.C., Chang H.Y. (2018). Epigenomics: Technologies and Applications. Circ. Res..

[6] Hasin Y., Seldin M., Lusis A. (2017). Multi-omics approaches to disease. Genome Biol..

[7] Hanahan D., Weinberg R.A. (2011). Hallmarks of cancer: The next generation. Cell.

[8] Chakraborty S., Hosen M.I., Ahmed M., Shekhar H.U. (2018). Onco-multi-OMICS approach: A new frontier in cancer research. Biomed. Res. Int..

[9] Tomczak K., Czerwinska P., Wiznerowicz M. (2015). The cancer genome atlas (TCGA): An immeasurable source of knowledge. Contemp. Oncol..

[10] Zhang J., Bajari R., Andric D., Gerthoffert F., Lepsa A., Nahal-Bose H., Stein L.D., Ferretti V. (2019). The International Cancer Genome Consortium Data Portal. Nat. Biotechnol..

[11] Morganti S., Tarantino P., Ferraro E., D’Amico P., Viale G., Trapani D., Duso B.A., Curigliano G. (2019). Complexity of genome sequencing and reporting: Next generation sequencing (NGS) technologies and implementation of precision medicine in real life. Crit. Rev. Oncol. Hematol..

[12] Shendure J., Balasubramanian S., Church G.M., Gilbert W., Rogers J., Schloss J.A., Waterston R.H. (2017). DNA sequencing at 40: Past, present and future. Nature.

[13] Del Giacco L., Cattaneo C. (2012). Introduction to genomics. Methods Mol. Biol..

[14] Pareek C.S., Smoczynski R., Tretyn A. (2011). Sequencing technologies and genome sequencing. J. Appl. Genet..

[15] Berger M.F., Mardis E.R. (2018). The emerging clinical relevance of genomics in cancer medicine. Nat. Rev. Clin. Oncol..

[16] Haley B., Roudnicky F. (2020). Functional genomics for cancer drug target discovery. Cancer Cell.

[17] Farid S.G., Morris-Stiff G. (2015). “OMICS” technologies and their role in foregut primary malignancies. Curr. Probl. Surg..

[18] Stratton M.R. (2011). Exploring the genomes of cancer cells: Progress and promise. Science.

[19] Zhang J., Wu G., Miller C.P., Tatevossian R.G., Dalton J.D., Tang B., Orisme W., Punchihewa C., Parker M., Qaddoumi I. (2013). Whole-genome sequencing identifies genetic alterations in pediatric low-grade gliomas. Nat. Genet..

[20] Zhu G., Benayed R., Ho C., Mullaney K., Sukhadia P., Rios K., Berry R., Rubin B.P., Nafa K., Wang L. (2019). Diagnosis of known sarcoma fusions and novel fusion partners by targeted RNA sequencing with identification of a recurrent ACTB-FOSB fusion in pseudomyogenic hemangioendothelioma. Mod. Pathol..

[21] Stangl C., de Blank S., Renkens I., Westera L., Verbeek T., Valle-Inclan J.E., Gonzalez R.C., Henssen A.G., van Roosmalen M.J., Stam R.W. (2020). Partner independent fusion gene detection by multiplexed CRISPR-Cas9 enrichment and long read nanopore sequencing. Nat. Commun..

[22] Schulze K., Imbeaud S., Letouze E., Alexandrov L.B., Calderaro J., Rebouissou S., Couchy G., Meiller C., Shinde J., Soysouvanh F. (2015). Exome sequencing of hepatocellular carcinomas identifies new mutational signatures and potential therapeutic targets. Nat. Genet..

[23] Choi J.H., Li Y., Guo J., Pei L., Rauch T.A., Kramer R.S., Macmil S.L., Wiley G.B., Bennett L.B., Schnabel J.L. (2010). Genome-wide DNA methylation maps in follicular lymphoma cells determined by methylation-enriched bisulfite sequencing. PLoS ONE.

[24] Gilpatrick T., Lee I., Graham J.E., Raimondeau E., Bowen R., Heron A., Downs B., Sukumar S., Sedlazeck F.J., Timp W. (2020). Targeted nanopore sequencing with Cas9-guided adapter ligation. Nat. Biotechnol..

[25] Kim H.J., Moon S.J., Hong S., Won H.H., Kim J.H. (2022). DBC1 is a key positive regulator of enhancer epigenomic writers KMT2D and p300. Nucleic Acids Res..

[26] Levin J.Z., Berger M.F., Adiconis X., Rogov P., Melnikov A., Fennell T., Nusbaum C., Garraway L.A., Gnirke A. (2009). Targeted next-generation sequencing of a cancer transcriptome enhances detection of sequence variants and novel fusion transcripts. Genome Biol..

[27] Hoogstrate Y., Komor M.A., Bottcher R., van Riet J., van de Werken H.J.G., van Lieshout S., Hoffmann R., van den Broek E., Bolijn A.S., Dits N. (2021). Fusion transcripts and their genomic breakpoints in polyadenylated and ribosomal RNA-minus RNA sequencing data. Gigascience.

[28] Qu H., Wang Z., Zhang Y., Zhao B., Jing S., Zhang J., Ye C., Xue Y., Yang L. (2022). Long-read nanopore sequencing identifies mismatch repair-deficient related genes with alternative splicing in colorectal cancer. Dis. Markers.

[29] Zhang S., Zhang X., Lei W., Liang J., Xu Y., Liu H., Ma S. (2019). Genome-wide profiling reveals alternative polyadenylation of mRNA in human non-small cell lung cancer. J. Transl. Med..

[30] Rabbani B., Tekin M., Mahdieh N. (2014). The promise of whole-exome sequencing in medical genetics. J. Hum. Genet..

[31] Lawrence M.S., Stojanov P., Polak P., Kryukov G.V., Cibulskis K., Sivachenko A., Carter S.L., Stewart C., Mermel C.H., Roberts S.A. (2013). Mutational heterogeneity in cancer and the search for new cancer-associated genes. Nature.

[32] Kozarewa I., Armisen J., Gardner A.F., Slatko B.E., Hendrickson C.L. (2015). Overview of target enrichment strategies. Curr. Protoc. Mol. Biol..

[33] Sjoblom T., Jones S., Wood L.D., Parsons D.W., Lin J., Barber T.D., Mandelker D., Leary R.J., Ptak J., Silliman N. (2006). The consensus coding sequences of human breast and colorectal cancers. Science.

[34] Vanlallawma A., Lallawmzuali D., Pautu J.L., Scaria V., Sivasubbu S., Kumar N.S. (2022). Whole exome sequencing of pediatric leukemia reveals a novel InDel within FLT-3 gene in AML patient from Mizo tribal population, Northeast India. BMC Genom. Data.

[35] Fewings E., Larionov A., Redman J., Goldgraben M.A., Scarth J., Richardson S., Brewer C., Davidson R., Ellis I., Evans D.G. (2018). Germline pathogenic variants in PALB2 and other cancer-predisposing genes in families with hereditary diffuse gastric cancer without CDH1 mutation: A whole-exome sequencing study. Lancet Gastroenterol. Hepatol..

[36] Manier S., Park J., Capelletti M., Bustoros M., Freeman S.S., Ha G., Rhoades J., Liu C.J., Huynh D., Reed S.C. (2018). Whole-exome sequencing of cell-free DNA and circulating tumor cells in multiple myeloma. Nat. Commun..

[37] Rheinbay E., Nielsen M.M., Abascal F., Wala J.A., Shapira O., Tiao G., Hornshoj H., Hess J.M., Juul R.I., Lin Z. (2020). Analyses of non-coding somatic drivers in 2658 cancer whole genomes. Nature.

[38] Hofmann A.L., Behr J., Singer J., Kuipers J., Beisel C., Schraml P., Moch H., Beerenwinkel N. (2017). Detailed simulation of cancer exome sequencing data reveals differences and common limitations of variant callers. BMC Bioinform..

[39] Royer-Bertrand B., Cisarova K., Niel-Butschi F., Mittaz-Crettol L., Fodstad H., Superti-Furga A. (2021). CNV detection from exome sequencing data in routine diagnostics of rare genetic disorders: Opportunities and limitations. Genes.

[40] Bewicke-Copley F., Arjun Kumar E., Palladino G., Korfi K., Wang J. (2019). Applications and analysis of targeted genomic sequencing in cancer studies. Comput. Struct. Biotechnol. J..

[41] Schultzhaus Z., Wang Z., Stenger D. (2021). CRISPR-based enrichment strategies for targeted sequencing. Biotechnol. Adv..

[42] Nagahashi M., Shimada Y., Ichikawa H., Kameyama H., Takabe K., Okuda S., Wakai T. (2019). Next generation sequencing-based gene panel tests for the management of solid tumors. Cancer Sci..

[43] Jennings L.J., Arcila M.E., Corless C., Kamel-Reid S., Lubin I.M., Pfeifer J., Temple-Smolkin R.L., Voelkerding K.V., Nikiforova M.N. (2017). guidelines for validation of next-generation sequencing-based oncology panels: A joint consensus recommendation of the association for molecular pathology and college of american pathologists. J. Mol. Diagn..

[44] Chang Y.S., Huang H.D., Yeh K.T., Chang J.G. (2017). Identification of novel mutations in endometrial cancer patients by whole-exome sequencing. Int. J. Oncol..

[45] Chang Y.S., Huang H.D., Yeh K.T., Chang J.G. (2016). Genetic alterations in endometrial cancer by targeted next-generation sequencing. Exp. Mol. Pathol..

[46] Dulak A.M., Stojanov P., Peng S., Lawrence M.S., Fox C., Stewart C., Bandla S., Imamura Y., Schumacher S.E., Shefler E. (2013). Exome and whole-genome sequencing of esophageal adenocarcinoma identifies recurrent driver events and mutational complexity. Nat. Genet..

[47] Heydt C., Wolwer C.B., Velazquez Camacho O., Wagener-Ryczek S., Pappesch R., Siemanowski J., Rehker J., Haller F., Agaimy A., Worm K. (2021). Detection of gene fusions using targeted next-generation sequencing: A comparative evaluation. BMC Med. Genom..

[48] Park H.J., Baek I., Cheang G., Solomon J.P., Song W. (2021). Comparison of RNA-based next-generation sequencing assays for the detection of NTRK gene fusions. J. Mol. Diagn..

[49] Miller D.E., Sulovari A., Wang T., Loucks H., Hoekzema K., Munson K.M., Lewis A.P., Fuerte E.P.A., Paschal C.R., Walsh T. (2021). Targeted long-read sequencing identifies missing disease-causing variation. Am. J. Hum. Genet..

[50] Jeong M., Guzman A.G., Goodell M.A. (2017). Genome-Wide Analysis of DNA Methylation in hematopoietic cells: DNA methylation analysis by WGBS. Methods Mol. Biol..

[51] Nakabayashi K., Yamamura M., Haseagawa K., Hata K. (2022). Reduced representation bisulfite sequencing (RRBS). Methods Mol. Biol..

[52] Kirschner K., Krueger F., Green A.R., Chandra T. (2018). Multiplexing for oxidative bisulfite sequencing (oxBS-seq). Methods Mol. Biol..

[53] Yu M., Han D., Hon G.C., He C. (2018). Tet-assisted bisulfite sequencing (TAB-seq). Methods Mol. Biol..

[54] Song C.X., Szulwach K.E., Dai Q., Fu Y., Mao S.Q., Lin L., Street C., Li Y., Poidevin M., Wu H. (2013). Genome-wide profiling of 5-formylcytosine reveals its roles in epigenetic priming. Cell.

[55] Lu X., Song C.X., Szulwach K., Wang Z., Weidenbacher P., Jin P., He C. (2013). Chemical modification-assisted bisulfite sequencing (CAB-Seq) for 5-carboxylcytosine detection in DNA. J. Am. Chem. Soc..

[56] Taiwo O., Wilson G.A., Morris T., Seisenberger S., Reik W., Pearce D., Beck S., Butcher L.M. (2012). Methylome analysis using MeDIP-seq with low DNA concentrations. Nat. Protoc..

[57] Lan X., Adams C., Landers M., Dudas M., Krissinger D., Marnellos G., Bonneville R., Xu M., Wang J., Huang T.H. (2011). High resolution detection and analysis of CpG dinucleotides methylation using MBD-Seq technology. PLoS ONE.

[58] Li D., Zhang B., Xing X., Wang T. (2015). Combining MeDIP-seq and MRE-seq to investigate genome-wide CpG methylation. Methods.

[59] Flusberg B.A., Webster D.R., Lee J.H., Travers K.J., Olivares E.C., Clark T.A., Korlach J., Turner S.W. (2010). Direct detection of DNA methylation during single-molecule, real-time sequencing. Nat. Methods.

[60] Tse O.Y.O., Jiang P., Cheng S.H., Peng W., Shang H., Wong J., Chan S.L., Poon L.C.Y., Leung T.Y., Chan K.C.A. (2021). Genome-wide detection of cytosine methylation by single molecule real-time sequencing. Proc. Natl. Acad. Sci. USA.

[61] Pai S.S., Ranjan S., Mathew A.R., Anindya R., Meur G. (2022). Analysis of the long-read sequencing data using computational tools confirms the presence of 5-methylcytosine in the Saccharomyces cerevisiae genome. Access Microbiol..

[62] Zhang J., Xie S., Xu J., Liu H., Wan S. (2021). Cancer biomarkers discovery of methylation modification with direct high-throughput nanopore sequencing. Front. Genet..

[63] Park P.J. (2009). ChIP-seq: Advantages and challenges of a maturing technology. Nat. Rev. Genet..

[64] Hainer S.J., Fazzio T.G. (2019). High-resolution chromatin profiling using CUT&RUN. Curr. Protoc. Mol. Biol..

[65] Kaya-Okur H.S., Wu S.J., Codomo C.A., Pledger E.S., Bryson T.D., Henikoff J.G., Ahmad K., Henikoff S. (2019). CUT&Tag for efficient epigenomic profiling of small samples and single cells. Nat. Commun..

[66] Song L., Crawford G.E. (2010). DNase-seq: A high-resolution technique for mapping active gene regulatory elements across the genome from mammalian cells. Cold Spring Harb. Protoc..

[67] Davie K., Jacobs J., Atkins M., Potier D., Christiaens V., Halder G., Aerts S. (2015). Discovery of transcription factors and regulatory regions driving in vivo tumor development by ATAC-seq and FAIRE-seq open chromatin profiling. PLoS Genet..

[68] Rizzo J.M., Sinha S. (2014). Analyzing the global chromatin structure of keratinocytes by MNase-seq. Methods Mol. Biol..

[69] Belton J.M., McCord R.P., Gibcus J.H., Naumova N., Zhan Y., Dekker J. (2012). Hi-C: A comprehensive technique to capture the conformation of genomes. Methods.

[70] Li G., Fullwood M.J., Xu H., Mulawadi F.H., Velkov S., Vega V., Ariyaratne P.N., Mohamed Y.B., Ooi H.S., Tennakoon C. (2010). ChIA-PET tool for comprehensive chromatin interaction analysis with paired-end tag sequencing. Genome Biol..

[71] Hrdlickova R., Toloue M., Tian B. (2017). RNA-Seq methods for transcriptome analysis. Wiley Interdiscip. Rev. RNA.

[72] Heidrich N., Bauriedl S., Schoen C. (2019). Investigating RNA-protein interactions in neisseria meningitidis by RIP-Seq analysis. Methods Mol. Biol..

[73] Darnell R.B. (2010). HITS-CLIP: Panoramic views of protein-RNA regulation in living cells. Wiley Interdiscip. Rev. RNA.

[74] Garzia A., Morozov P., Sajek M., Meyer C., Tuschl T. (2018). PAR-CLIP for discovering target sites of RNA-binding proteins. Methods Mol. Biol..

[75] Helwak A., Tollervey D. (2014). Mapping the miRNA interactome by cross-linking ligation and sequencing of hybrids (CLASH). Nat. Protoc..

[76] Sharma E., Sterne-Weiler T., O’Hanlon D., Blencowe B.J. (2016). Global Mapping of human RNA-RNA interactions. Mol. Cell.

[77] Chu C., Quinn J., Chang H.Y. (2012). Chromatin isolation by RNA purification (ChIRP). J. Vis. Exp..

[78] Simon M.D. (2013). Capture hybridization analysis of RNA targets (CHART). Curr. Protoc. Mol. Biol..

[79] Zhou B., Li X., Luo D., Lim D.H., Zhou Y., Fu X.D. (2019). GRID-seq for comprehensive analysis of global RNA-chromatin interactions. Nat. Protoc..

[80] Jukam D., Limouse C., Smith O.K., Risca V.I., Bell J.C., Straight A.F. (2019). Chromatin-Associated RNA Sequencing (ChAR-seq). Curr. Protoc. Mol. Biol..

[81] Watters K.E., Yu A.M., Strobel E.J., Settle A.H., Lucks J.B. (2016). Characterizing RNA structures in vitro and in vivo with selective 2′-hydroxyl acylation analyzed by primer extension sequencing (SHAPE-Seq). Methods.

[82] Siegfried N.A., Busan S., Rice G.M., Nelson J.A., Weeks K.M. (2014). RNA motif discovery by SHAPE and mutational profiling (SHAPE-MaP). Nat. Methods.

[83] Chen L., Chang H.Y., Artandi S.E. (2021). Analysis of RNA conformation in endogenously assembled RNPs by icSHAPE. STAR Protoc..

[84] Wang Y., Zheng D., Tan Q., Wang M.X., Gu L.Q. (2011). Nanopore-based detection of circulating microRNAs in lung cancer patients. Nat. Nanotechnol..

[85] Gu L.Q., Wanunu M., Wang M.X., McReynolds L., Wang Y. (2012). Detection of miRNAs with a nanopore single-molecule counter. Expert Rev. Mol. Diagn..

[86] Zhang J., Yan S., Chang L., Guo W., Wang Y., Wang Y., Zhang P., Chen H.Y., Huang S. (2020). Direct microRNA sequencing Using nanopore-induced phase-shift sequencing. iScience.

[87] Van Dijk E.L., Jaszczyszyn Y., Naquin D., Thermes C. (2018). The third revolution in sequencing technology. Trends Genet..

[88] Sakamoto Y., Sereewattanawoot S., Suzuki A. (2020). A new era of long-read sequencing for cancer genomics. J. Hum. Genet..

[89] Amarasinghe S.L., Su S., Dong X., Zappia L., Ritchie M.E., Gouil Q. (2020). Opportunities and challenges in long-read sequencing data analysis. Genome Biol..

[90] Bai X., Li Y., Zeng X., Zhao Q., Zhang Z. (2021). Single-cell sequencing technology in tumor research. Clin. Chim. Acta.

[91] Huang L., Ma F., Chapman A., Lu S., Xie X.S. (2015). Single-cell whole-genome amplification and sequencing: Methodology and applications. Annu. Rev. Genom. Hum. Genet..

[92] Yasen A., Aini A., Wang H., Li W., Zhang C., Ran B., Tuxun T., Maimaitinijiati Y., Shao Y., Aji T. (2020). Progress and applications of single-cell sequencing techniques. Infect. Genet. Evol..

[93] Sanders A.D., Falconer E., Hills M., Spierings D.C.J., Lansdorp P.M. (2017). Single-cell template strand sequencing by Strand-seq enables the characterization of individual homologs. Nat. Protoc..

[94] Rivera C.M., Ren B. (2013). Mapping human epigenomes. Cell.

[95] Li Y. (2021). Modern epigenetics methods in biological research. Methods.

[96] Nebbioso A., Tambaro F.P., Dell’Aversana C., Altucci L. (2018). Cancer epigenetics: Moving forward. PLoS Genet..

[97] Bohnsack K.E., Hobartner C., Bohnsack M.T. (2019). Eukaryotic 5-methylcytosine (m^5^C) RNA methyltransferases: Mechanisms, cellular functions, and links to disease. Genes.

[98] Sarda S., Hannenhalli S. (2018). Orphan CpG islands as alternative promoters. Transcription.

[99] Joo J.E., Dowty J.G., Milne R.L., Wong E.M., Dugue P.A., English D., Hopper J.L., Goldgar D.E., Giles G.G., Southey M.C. (2018). Heritable DNA methylation marks associated with susceptibility to breast cancer. Nat. Commun..

[100] Usui G., Matsusaka K., Mano Y., Urabe M., Funata S., Fukayama M., Ushiku T., Kaneda A. (2021). DNA methylation and genetic aberrations in gastric cancer. Digestion.

[101] Tse J.W.T., Jenkins L.J., Chionh F., Mariadason J.M. (2017). Aberrant DNA methylation in colorectal cancer: What should we target?. Trends Cancer.

[102] Zafon C., Gil J., Perez-Gonzalez B., Jorda M. (2019). DNA methylation in thyroid cancer. Endocr. Relat. Cancer.

[103] Beaulaurier J., Schadt E.E., Fang G. (2019). Deciphering bacterial epigenomes using modern sequencing technologies. Nat. Rev. Genet..

[104] Darst R.P., Pardo C.E., Ai L., Brown K.D., Kladde M.P. (2010). Bisulfite sequencing of DNA. Curr. Protoc. Mol. Biol..

[105] Feng Z., Fang G., Korlach J., Clark T., Luong K., Zhang X., Wong W., Schadt E. (2013). Detecting DNA modifications from SMRT sequencing data by modeling sequence context dependence of polymerase kinetic. PLoS Comput. Biol..

[106] Wallace E.V., Stoddart D., Heron A.J., Mikhailova E., Maglia G., Donohoe T.J., Bayley H. (2010). Identification of epigenetic DNA modifications with a protein nanopore. Chem. Commun..

[107] Li Q.N., Guo L., Hou Y., Ou X.H., Liu Z., Sun Q.Y. (2018). The DNA methylation profile of oocytes in mice with hyperinsulinaemia and hyperandrogenism as detected by single-cell level whole genome bisulphite sequencing (SC-WGBS) technology. Reprod. Fertil. Dev..

[108] Audia J.E., Campbell R.M. (2016). Histone modifications and cancer. Cold Spring Harb. Perspect. Biol..

[109] Wang L., Gao Y., Zheng X., Liu C., Dong S., Li R., Zhang G., Wei Y., Qu H., Li Y. (2019). Histone modifications regulate chromatin compartmentalization by contributing to a phase separation mechanism. Mol. Cell.

[110] Morin R.D., Mendez-Lago M., Mungall A.J., Goya R., Mungall K.L., Corbett R.D., Johnson N.A., Severson T.M., Chiu R., Field M. (2011). Frequent mutation of histone-modifying genes in non-Hodgkin lymphoma. Nature.

[111] Grosselin K., Durand A., Marsolier J., Poitou A., Marangoni E., Nemati F., Dahmani A., Lameiras S., Reyal F., Frenoy O. (2019). High-throughput single-cell ChIP-seq identifies heterogeneity of chromatin states in breast cancer. Nat. Genet..

[112] Lareau C.A., Duarte F.M., Chew J.G., Kartha V.K., Burkett Z.D., Kohlway A.S., Pokholok D., Aryee M.J., Steemers F.J., Lebofsky R. (2019). Droplet-based combinatorial indexing for massive-scale single-cell chromatin accessibility. Nat. Biotechnol..

[113] Ai S., Xiong H., Li C.C., Luo Y., Shi Q., Liu Y., Yu X., Li C., He A. (2019). Profiling chromatin states using single-cell itChIP-seq. Nat. Cell Biol..

[114] Kumar S., Gonzalez E.A., Rameshwar P., Etchegaray J.P. (2020). Non-Coding RNAs as mediators of epigenetic changes in malignancies. Cancers.

[115] Bianchi M., Renzini A., Adamo S., Moresi V. (2017). Coordinated Actions of MicroRNAs with other epigenetic factors regulate skeletal muscle development and adaptation. Int. J. Mol. Sci..

[116] Wang K.C., Chang H.Y. (2011). Molecular mechanisms of long noncoding RNAs. Mol. Cell.

[117] Stark R., Grzelak M., Hadfield J. (2019). RNA sequencing: The teenage years. Nat. Rev. Genet..

[118] Giraldez M.D., Spengler R.M., Etheridge A., Godoy P.M., Barczak A.J., Srinivasan S., De Hoff P.L., Tanriverdi K., Courtright A., Lu S. (2018). Comprehensive multi-center assessment of small RNA-seq methods for quantitative miRNA profiling. Nat. Biotechnol..

[119] Hafner M., Landthaler M., Burger L., Khorshid M., Hausser J., Berninger P., Rothballer A., Ascano M., Jungkamp A.C., Munschauer M. (2010). Transcriptome-wide identification of RNA-binding protein and microRNA target sites by PAR-CLIP. Cell.

[120] McGettigan P.A. (2013). Transcriptomics in the RNA-seq era. Curr. Opin. Chem. Biol..

[121] Zhang G., Sun M., Wang J., Lei M., Li C., Zhao D., Huang J., Li W., Li S., Li J. (2019). PacBio full-length cDNA sequencing integrated with RNA-seq reads drastically improves the discovery of splicing transcripts in rice. Plant J..

[122] Ura H., Togi S., Niida Y. (2022). A comparison of mRNA sequencing (RNA-Seq) library preparation methods for transcriptome analysis. BMC Genom..

[123] Pyatnitskiy M.A., Arzumanian V.A., Radko S.P., Ptitsyn K.G., Vakhrushev I.V., Poverennaya E.V., Ponomarenko E.A. (2021). Oxford Nanopore MinION Direct RNA-Seq for Systems Biology. Biology.

[124] Neckles C., Sundara Rajan S., Caplen N.J. (2020). Fusion transcripts: Unexploited vulnerabilities in cancer?. Wiley Interdiscip. Rev. RNA.

[125] Kumar S., Razzaq S.K., Vo A.D., Gautam M., Li H. (2016). Identifying fusion transcripts using next generation sequencing. Wiley Interdiscip. Rev. RNA.

[126] Xu Z., Wang M., Shi D., Zhou G., Niu T., Hahn M.G., O’Neill M.A., Kong Y. (2017). DGE-seq analysis of MUR3-related Arabidopsis mutants provides insight into how dysfunctional xyloglucan affects cell elongation. Plant Sci..

[127] Wang M., Gu M., Liu L., Liu Y., Tian L. (2021). Single-cell RNA sequencing (scRNA-seq) in cardiac tissue: Applications and limitations. Vasc. Health Risk Manag..

[128] Zhao J., Jaffe A., Li H., Lindenbaum O., Sefik E., Jackson R., Cheng X., Flavell R.A., Kluger Y. (2021). Detection of differentially abundant cell subpopulations in scRNA-seq data. Proc. Natl. Acad. Sci. USA.

[129] Goetz J.J., Trimarchi J.M. (2012). Transcriptome sequencing of single cells with Smart-Seq. Nat. Biotechnol..

[130] Singh M., Al-Eryani G., Carswell S., Ferguson J.M., Blackburn J., Barton K., Roden D., Luciani F., Giang Phan T., Junankar S. (2019). High-throughput targeted long-read single cell sequencing reveals the clonal and transcriptional landscape of lymphocytes. Nat. Commun..

[131] Sarkar A., Gasperi W., Begley U., Nevins S., Huber S.M., Dedon P.C., Begley T.J. (2021). Detecting the epitranscriptome. Wiley Interdiscip. Rev. RNA.

[132] Moshitch-Moshkovitz S., Dominissini D., Rechavi G. (2022). The epitranscriptome toolbox. Cell.

[133] Nachtergaele S., He C. (2018). Chemical Modifications in the Life of an mRNA Transcript. Annu. Rev. Genet..

[134] Nombela P., Miguel-Lopez B., Blanco S. (2021). The role of m^6^A, m^5^C and Psi RNA modifications in cancer: Novel therapeutic opportunities. Mol. Cancer.

[135] Barbieri I., Kouzarides T. (2020). Role of RNA modifications in cancer. Nat. Rev. Cancer.

[136] Lin S., Choe J., Du P., Triboulet R., Gregory R.I. (2016). The m^6^A methyltransferase METTL3 promotes translation in human cancer cells. Mol. Cell.

[137] Liu J., Eckert M.A., Harada B.T., Liu S.M., Lu Z., Yu K., Tienda S.M., Chryplewicz A., Zhu A.C., Yang Y. (2018). m^6^A mRNA methylation regulates AKT activity to promote the proliferation and tumorigenicity of endometrial cancer. Nat. Cell Biol..

[138] Liu T., Hu X., Lin C., Shi X., He Y., Zhang J., Cai K. (2022). 5-methylcytosine RNA methylation regulators affect prognosis and tumor microenvironment in lung adenocarcinoma. Ann. Transl. Med..

[139] Frye M., Watt F.M. (2006). The RNA methyltransferase Misu (NSun2) mediates Myc-induced proliferation and is upregulated in tumors. Curr. Biol..

[140] Woo H.H., Chambers S.K. (2019). Human ALKBH3-induced m^1^A demethylation increases the CSF-1 mRNA stability in breast and ovarian cancer cells. Biochim. Biophys. Acta Gene Regul. Mech..

[141] Garus A., Autexier C. (2021). Dyskerin: An essential pseudouridine synthase with multifaceted roles in ribosome biogenesis, splicing, and telomere maintenance. RNA.

[142] Martinez N.M., Su A., Burns M.C., Nussbacher J.K., Schaening C., Sathe S., Yeo G.W., Gilbert W.V. (2022). Pseudouridine synthases modify human pre-mRNA co-transcriptionally and affect pre-mRNA processing. Mol. Cell.

[143] Li H., Chen L., Han Y., Zhang F., Wang Y., Han Y., Wang Y., Wang Q., Guo X. (2021). The Identification of RNA Modification Gene PUS7 as a Potential Biomarker of Ovarian Cancer. Biology.

[144] Song J., Zhuang Y., Zhu C., Meng H., Lu B., Xie B., Peng J., Li M., Yi C. (2020). Differential roles of human PUS10 in miRNA processing and tRNA pseudouridylation. Nat. Chem. Biol..

[145] Jana S., Hsieh A.C., Gupta R. (2017). Reciprocal amplification of caspase-3 activity by nuclear export of a putative human RNA-modifying protein, PUS10 during TRAIL-induced apoptosis. Cell Death Dis..

[146] Dominissini D., Moshitch-Moshkovitz S., Amariglio N., Rechavi G. (2011). Adenosine-to-inosine RNA editing meets cancer. Carcinogenesis.

[147] Chen L., Li Y., Lin C.H., Chan T.H., Chow R.K., Song Y., Liu M., Yuan Y.F., Fu L., Kong K.L. (2013). Recoding RNA editing of AZIN1 predisposes to hepatocellular carcinoma. Nat. Med..

[148] Wetzel C., Limbach P.A. (2016). Mass spectrometry of modified RNAs: Recent developments. Analyst.

[149] Giessing A.M., Kirpekar F. (2012). Mass spectrometry in the biology of RNA and its modifications. J. Proteom..

[150] Li X., Xiong X., Yi C. (2016). Epitranscriptome sequencing technologies: Decoding RNA modifications. Nat. Methods.

[151] Meyer K.D. (2019). DART-seq: An antibody-free method for global m^6^A detection. Nat. Methods.

[152] Wang D.O. (2019). Mapping m^6^A and m^1^A with mutation signatures. Nat. Methods.

[153] Gu X., Liang Z. (2019). Transcriptome-Wide Mapping 5-Methylcytosine by m^5^C RNA Immunoprecipitation Followed by Deep Sequencing in Plant. Methods Mol. Biol..

[154] Li W., Li X., Ma X., Xiao W., Zhang J. (2022). Mapping the m1A, m5C, m6A and m7G methylation atlas in zebrafish brain under hypoxic conditions by MeRIP-seq. BMC Genom..

[155] Khoddami V., Cairns B.R. (2014). Transcriptome-wide target profiling of RNA cytosine methyltransferases using the mechanism-based enrichment procedure Aza-IP. Nat. Protoc..

[156] Carlile T.M., Rojas-Duran M.F., Gilbert W.V. (2015). Transcriptome-wide identification of pseudouridine modifications using pseudo-seq. Curr. Protoc. Mol. Biol..

[157] Bruno R., Fontanini G. (2020). Next generation sequencing for gene fusion analysis in lung cancer: A literature review. Diagnostics.

[158] Dacic S., Villaruz L.C., Abberbock S., Mahaffey A., Incharoen P., Nikiforova M.N. (2016). ALK FISH patterns and the detection of ALK fusions by next generation sequencing in lung adenocarcinoma. Oncotarget.

[159] Vollbrecht C., Lenze D., Hummel M., Lehmann A., Moebs M., Frost N., Jurmeister P., Schweizer L., Kellner U., Dietel M. (2018). RNA-based analysis of ALK fusions in non-small cell lung cancer cases showing IHC/FISH discordance. BMC Cancer.

[160] Zhao L., Zhang H., Kohnen M.V., Prasad K., Gu L., Reddy A.S.N. (2019). Analysis of transcriptome and epitranscriptome in plants using pacbio Iso-seq and nanopore-based direct RNA SEQUENCING. Front. Genet..

[161] Wang Z., Gerstein M., Snyder M. (2009). RNA-Seq: A revolutionary tool for transcriptomics. Nat. Rev. Genet..

[162] Gildea M.A., Dwyer Z.W., Pleiss J.A. (2020). Multiplexed primer extension sequencing: A targeted RNA-seq method that enables high-precision quantitation of mRNA splicing isoforms and rare pre-mRNA splicing intermediates. Methods.

[163] Byrne A., Beaudin A.E., Olsen H.E., Jain M., Cole C., Palmer T., DuBois R.M., Forsberg E.C., Akeson M., Vollmers C. (2017). Nanopore long-read RNAseq reveals widespread transcriptional variation among the surface receptors of individual B cells. Nat. Commun..

[164] Beck A.H., Weng Z., Witten D.M., Zhu S., Foley J.W., Lacroute P., Smith C.L., Tibshirani R., van de Rijn M., Sidow A. (2010). 3′-end sequencing for expression quantification (3SEQ) from archival tumor samples. PLoS ONE.

[165] Chen W., Jia Q., Song Y., Fu H., Wei G., Ni T. (2017). Alternative Polyadenylation: Methods, Findings, and Impacts. Genom. Proteom. Bioinform..

[166] Harrison P.F., Powell D.R., Clancy J.L., Preiss T., Boag P.R., Traven A., Seemann T., Beilharz T.H. (2015). PAT-seq: A method to study the integration of 3′-UTR dynamics with gene expression in the eukaryotic transcriptome. RNA.

[167] Routh A., Ji P., Jaworski E., Xia Z., Li W., Wagner E.J. (2017). Poly(A)-ClickSeq: Click-chemistry for next-generation 3′-end sequencing without RNA enrichment or fragmentation. Nucleic Acids Res..

[168] Anvar S.Y., Allard G., Tseng E., Sheynkman G.M., de Klerk E., Vermaat M., Yin R.H., Johansson H.E., Ariyurek Y., den Dunnen J.T. (2018). Full-length mRNA sequencing uncovers a widespread coupling between transcription initiation and mRNA processing. Genome Biol..

[169] Legnini I., Alles J., Karaiskos N., Ayoub S., Rajewsky N. (2019). FLAM-seq: Full-length mRNA sequencing reveals principles of poly(A) tail length control. Nat. Methods.

[170] Di Bella S., La Ferlita A., Carapezza G., Alaimo S., Isacchi A., Ferro A., Pulvirenti A., Bosotti R. (2020). A benchmarking of pipelines for detecting ncRNAs from RNA-Seq data. Brief. Bioinform..

[171] Chen C.J., Servant N., Toedling J., Sarazin A., Marchais A., Duvernois-Berthet E., Cognat V., Colot V., Voinnet O., Heard E. (2012). ncPRO-seq: A tool for annotation and profiling of ncRNAs in sRNA-seq data. Bioinformatics.

[172] Hu J.F., Yim D., Ma D., Huber S.M., Davis N., Bacusmo J.M., Vermeulen S., Zhou J., Begley T.J., DeMott M.S. (2021). Quantitative mapping of the cellular small RNA landscape with AQRNA-seq. Nat. Biotechnol..

[173] Motameny S., Wolters S., Nurnberg P., Schumacher B. (2010). Next Generation Sequencing of miRNAs—Strategies, Resources and Methods. Genes.

[174] Chen X., Chen L., Tang Y., He Y., Pan K., Yuan L., Xie W., Chen S., Zhao W., Yu D. (2022). Transcriptome-wide m^6^A methylome analysis uncovered the changes of m^6^A modification in oral pre-malignant cells compared with normal oral epithelial cells. Front. Oncol..

[175] Chen H.X., Zhang Z., Ma D.Z., Chen L.Q., Luo G.Z. (2022). Mapping single-nucleotide m^6^A by m^6^A-REF-seq. Methods.

[176] Leger A., Amaral P.P., Pandolfini L., Capitanchik C., Capraro F., Miano V., Migliori V., Toolan-Kerr P., Sideri T., Enright A.J. (2021). RNA modifications detection by comparative Nanopore direct RNA sequencing. Nat. Commun..

[177] Schaefer M. (2015). RNA 5-Methylcytosine Analysis by Bisulfite Sequencing. Methods Enzymol..

[178] Zhang S., Li R., Zhang L., Chen S., Xie M., Yang L., Xia Y., Foyer C.H., Zhao Z., Lam H.M. (2020). New insights into Arabidopsis transcriptome complexity revealed by direct sequencing of native RNAs. Nucleic Acids Res..

[179] Carlile T.M., Martinez N.M., Schaening C., Su A., Bell T.A., Zinshteyn B., Gilbert W.V. (2019). mRNA structure determines modification by pseudouridine synthase 1. Nat. Chem. Biol..

[180] Cozen A.E., Quartley E., Holmes A.D., Hrabeta-Robinson E., Phizicky E.M., Lowe T.M. (2015). ARM-seq: AlkB-facilitated RNA methylation sequencing reveals a complex landscape of modified tRNA fragments. Nat. Methods.

[181] Zhou H., Rauch S., Dai Q., Cui X., Zhang Z., Nachtergaele S., Sepich C., He C., Dickinson B.C. (2019). Evolution of a reverse transcriptase to map N^1^-methyladenosine in human messenger RNA. Nat. Methods.

[182] Safra M., Sas-Chen A., Nir R., Winkler R., Nachshon A., Bar-Yaacov D., Erlacher M., Rossmanith W., Stern-Ginossar N., Schwartz S. (2017). The m1A landscape on cytosolic and mitochondrial mRNA at single-base resolution. Nature.

[183] Zheng H.X., Zhang X.S., Sui N. (2020). Advances in the profiling of N^6^-methyladenosine (m^6^A) modifications. Biotechnol. Adv..

[184] Potapov V., Fu X., Dai N., Correa I.R., Tanner N.A., Ong J.L. (2018). Base modifications affecting RNA polymerase and reverse transcriptase fidelity. Nucleic Acids Res..

[185] Vilfan I.D., Tsai Y.C., Clark T.A., Wegener J., Dai Q., Yi C., Pan T., Turner S.W., Korlach J. (2013). Analysis of RNA base modification and structural rearrangement by single-molecule real-time detection of reverse transcription. J. Nanobiotechnology.

[186] Noakes M.T., Brinkerhoff H., Laszlo A.H., Derrington I.M., Langford K.W., Mount J.W., Bowman J.L., Baker K.S., Doering K.M., Tickman B.I. (2019). Increasing the accuracy of nanopore DNA sequencing using a time-varying cross membrane voltage. Nat. Biotechnol..

[187] Johnson D.T., Harris R.A., French S., Blair P.V., You J., Bemis K.G., Wang M., Balaban R.S. (2007). Tissue heterogeneity of the mammalian mitochondrial proteome. Am. J. Physiol. Cell Physiol..

[188] Shruthi B.S., Vinodhkumar P., Selvamani (2016). Proteomics: A new perspective for cancer. Adv. Biomed. Res..

[189] Collins B.C., Aebersold R. (2018). Proteomics goes parallel. Nat. Biotechnol..

[190] Restrepo-Perez L., Joo C., Dekker C. (2018). Paving the way to single-molecule protein sequencing. Nat. Nanotechnol..

[191] Mantini G., Pham T.V., Piersma S.R., Jimenez C.R. (2021). Computational analysis of phosphoproteomics data in Multi-Omics cancer studies. Proteomics.

[192] Tang L. (2018). Next-generation peptide sequencing. Nat. Methods.

[193] Swaminathan J., Boulgakov A.A., Hernandez E.T., Bardo A.M., Bachman J.L., Marotta J., Johnson A.M., Anslyn E.V., Marcotte E.M. (2018). Highly parallel single-molecule identification of proteins in zeptomole-scale mixtures. Nat. Biotechnol..

[194] Ouldali H., Sarthak K., Ensslen T., Piguet F., Manivet P., Pelta J., Behrends J.C., Aksimentiev A., Oukhaled A. (2020). Electrical recognition of the twenty proteinogenic amino acids using an aerolysin nanopore. Nat. Biotechnol..

[195] Wu W., Hu W., Kavanagh J.J. (2002). Proteomics in cancer research. Int. J. Gynecol. Cancer.

[196] Kwon Y.W., Jo H.S., Bae S., Seo Y., Song P., Song M., Yoon J.H. (2021). Application of proteomics in cancer: Recent trends and approaches for biomarkers discovery. Front. Med..

[197] Wheeler H.E., Maitland M.L., Dolan M.E., Cox N.J., Ratain M.J. (2013). Cancer pharmacogenomics: Strategies and challenges. Nat. Rev. Genet..

[198] Katsila T., Patrinos G.P. (2015). Whole genome sequencing in pharmacogenomics. Front. Pharmacol..

[199] Cerea G., Ricotta R., Schiavetto I., Maugeri M.R., Sartore-Bianchi A., Moroni M., Artale S., Siena S. (2006). Cetuximab for treatment of metastatic colorectal cancer. Ann. Oncol..

[200] Mondaca S., Lebow E.S., Namakydoust A., Razavi P., Reis-Filho J.S., Shen R., Offin M., Tu H.Y., Murciano-Goroff Y., Xu C. (2021). Clinical utility of next-generation sequencing-based ctDNA testing for common and novel ALK fusions. Lung Cancer.

[201] Onidani K., Shoji H., Kakizaki T., Yoshimoto S., Okaya S., Miura N., Sekikawa S., Furuta K., Lim C.T., Shibahara T. (2019). Monitoring of cancer patients via next-generation sequencing of patient-derived circulating tumor cells and tumor DNA. Cancer Sci..

